# Comparison of Interventions to Improve Long-Term Medication Adherence Across Different Clinical Conditions: A Systematic Review With Network Meta-Analysis

**DOI:** 10.3389/fphar.2018.01454

**Published:** 2018-12-24

**Authors:** Andrea Torres-Robles, Elyssa Wiecek, Fernanda S. Tonin, Shalom I. Benrimoj, Fernando Fernandez-Llimos, Victoria Garcia-Cardenas

**Affiliations:** ^1^Graduate School of Health, University of Technology Sydney, Sydney, NSW, Australia; ^2^Pharmaceutical Sciences Postgraduate Programme, Universidade Federal do Paraná, Curitiba, Brazil; ^3^Department of Social Pharmacy, Faculty of Pharmacy, Research Institute for Medicines (iMed.Ulisboa), Universidade de Lisboa, Lisbon, Portugal

**Keywords:** medication adherence, network meta-analysis, chronic diseases, long-term, intervention, adherence implementation

## Abstract

**Background:** Medication non-adherence has a dynamic, temporal and multifactorial nature with a significant impact on economic and clinical outcomes. Interventions to improve adherence are complex and require adaptation to patients' needs, which may include patient's medical conditions. The aim of this study was to assess the comparative effectiveness of medication adherence interventions per type of clinical condition on adult patients.

**Methods:** A systematic review with network meta-analysis was performed (PROSPERO registration number of CRD42018054598). An initial Pubmed search was conducted to select meta-analyses reporting results of interventions aiming to improve medication adherence. Primary studies were selected and those reporting results with a long-term follow up (≥10 months) on adult patients were included for data extraction. Study characteristics, description of interventions and adherence outcomes were extracted. Adherence interventions were classified in four groups: educational, attitudinal, technical, and rewards. Clinical conditions were classified in four groups: circulatory system and metabolic diseases, infectious diseases, musculoskeletal diseases, and mental, behavioral or neurodevelopmental disorders. Network meta-analyses with effect sizes expressed as odds ratio (OR) with a 95% credibility interval (CrI) were built. Ranking probabilities for each measure of adherence were calculated by using surface under the cumulative ranking analysis (SUCRA).

**Results:** A total of 61 meta-analysis and 149 primary studies were included in the qualitative synthesis and 80 primary studies in the quantitative analysis. The most effective interventions were: educational + technical 79.6% [OR: 0.44 (CrI: 0.26, 0.73)] and 73.3% [OR: 0.56 (0.36, 0.84)] in circulatory system and metabolic diseases and infectious diseases respectively. Attitudinal intervention had the greatest probability for musculoskeletal diseases of 92.3% in SUCRA [OR: 0.30 (0.10, 0.86)]. Finally, educational + attitudinal interventions had the greatest effect (SUCRA 73.8%) for mental, behavioral or neurodevelopmental disorders, although this was not significant according to consistency analysis.

**Conclusion:** Effectiveness of interventions seems to be related to the clinical condition. Educational and technical interventions resulted in a major effect on long-term management of medication adherence in patients with infectious diseases (HIV) and circulatory system and metabolic diseases whereas attitudinal components presented a higher effect on musculoskeletal and mental, behavioral or neurodevelopmental disorders.

## Introduction

Medication non-adherence represents a continuous burden for the health-care system. Statistics remain constant since 2003, when the World Health Organization reported at least 50% of patients with chronic conditions were non-adherent to their medications (Sabate, 2003). Non-adherence can occur at different stages during the course of therapy, including initiation, implementation and persistence (Vrijens et al., 2012). A study analyzing an electronic database of nearly 17,000 patients' dosing histories across different diseases states for 1 year (including osteoporosis, diabetes, hypertension, depression and HIV), revealed 4% patients never initiated their treatment, nearly 40% discontinued, and only 55% dosed correctly (Blaschke et al., 2012).

The negative consequences of this phenomenon have been widely reported in the literature. For example, a recent systematic review found the economic impact of non-adherence, including the healthcare costs, ranged from $949 to $44,190 per patient annually across 14 disease groups (Cutler et al., 2018).

During the past 10 years there has been mounting evidence demonstrating the impact of diverse interventions on medication adherence in a range of clinical outcomes (Nieuwlaat et al., 2014). Effective adherence interventions have resulted in viral suppression in HIV patients (Mills et al., 2014), decrease of lipid levels and total cholesterol in patients taking lipid lowering medications (Deichmann et al., 2016), reduction of HbAc1, decrease hospitalizations and all-cause mortality in patients with diabetes (Ho et al., 2006), and reduction of risk of death and hospitalizations in patients with heart failure (Fitzgerald et al., 2011). Despite their proven efficacy, there is still a lack of consistent evidence on the core elements these interventions should include, limiting their implementation in routine practice. Effective interventions appear to be complex (through a combination of multiple core components) and tailored to the patient's needs (Nieuwlaat et al., 2014; Conn et al., 2016). Different intervention's success may be linked to the clinical condition being targeted. For example, there is some evidence technical interventions are effective in patients with hypertension (Conn et al., 2015), whereas interventions aiming to modify patients' beliefs and attitudes have been found to be more effective in patients with mental disorders (MacDonald et al., 2016; Readdean et al., 2018).

Heterogeneity of interventions and adherence measures is often reported to be a barrier for the quantitative analysis of interventions, hindering the comparison across different studies (Nieuwlaat et al., 2014). Some meta-analyses have overcome this limitation by directly comparing the effect of interventions on a range of adherence measures (Conn and Ruppar, 2017). However, these analyses lack indirect comparisons that could strengthen the current evidence. The use of network meta-analysis provides an advantage when compared to traditional meta-analysis methods, as it allows a comparison of multiple treatments or interventions at the same time, using both direct comparisons within randomized controlled trials and indirect comparisons across trials based on a common comparator (Tonin et al., 2017). Currently, a few network meta-analyses have been undertaken with the objective of assessing the impact of adherence interventions in HIV patients (Mills et al., 2014; Kanters et al., 2017).

Thus, the aim of this systematic review and network meta-analysis was to assess the comparative effectiveness of medication adherence interventions per type of clinical condition on adult patients being prescribed medications for the following condition groups: circulatory system and metabolic diseases, infectious diseases, musculoskeletal diseases, and mental, behavioral or neurodevelopmental disorders.

## Methods

As part of a larger project, this systematic review and network meta-analysis was performed following the Cochrane recommendations (Higgins JPT, 2011) and PRISMA statement for reporting systematic reviews incorporating network meta-analyses (Hutton et al., 2015) on health care interventions (PROSPERO registration number of CRD42018054598).

### Search Strategy and Eligibility Criteria

To avoid inefficient duplication of efforts in a field like medication adherence with a vast body of primary and secondary literature, a two-steps approach was used for literature selection (Nieuwlaat et al., 2014). The first step aimed to retrieve pairwise meta-analyses assessing interventions to improve medication adherence on adult patients. In a second step, primary articles identified in the meta-analyses reporting experimental controlled trials were identified as data sources for our study.

The meta-analyses were systematically searched in PubMed, which comprises Medline and PubMed Central, in October 2017 with no restriction on publication date or language. A first screening by title and abstract of the meta-analyses was performed by two independent investigators and discrepancies were solved by a third reviewer. The search strategy can be found on the Supplementary Material 1.

In the second step, primary studies were selected from the identified meta-analyses and were full-text reviewed by two investigators. Primary studies with an experimental controlled design (randomized or non-randomized clinical trials) assessing the long-term effect of adherence interventions (follow-up of more than 10 months) and reporting measures of adherence (i.e., self-repot, pill count, refill data, electronic monitoring) on adult patients with prescribed medications were included for data extraction. Studies were excluded if the interventions were not patient-focused, assessed adherence to the following medications (over the counter medications, depot medications, vaccines), were not written in Roman characters, or were unpublished studies (e.g., conference posters, dissertations). From the eligible studies, those reporting adherence results as a categorical variable were included in the network meta-analysis. Studies reporting continuous data were only considered for qualitative analysis. Other studies not included in the network meta-analysis were those with the same intervention in all the study arms (same comparator) and clinical conditions without a sufficient number of studies to perform a comparative analysis. Additional information regarding inclusion or exclusion criteria can be found in Supplementary Material 2.

### Data Extraction and Quality Assessment

Data from primary studies was extracted by two investigators and recorded on a standard data collection form. This included: authors, year of publication, country, sample size, clinical condition being targeted, sex, age, patient follow up period, study arms, interventions assessed, and measures of adherence. Targeted diseases were identified for each study and then classified in groups based on the International Classification of Diseases 11th Revision (ICD-11) (World Health Organization, 2018) into circulatory system and metabolic diseases, infectious diseases, musculoskeletal diseases, and mental, behavioral or neurodevelopmental disorders as described in Table 1. Circulatory system and metabolic diseases were classified as one group as they share common risk factors and patients are usually prescribed with medications from both groups (Cheung and Li, 2012).

**Table 1:** Definition of groups for classification of clinical conditions.

**Disease group**	**Clinical conditions included**
Circulatory system and metabolic diseases	Hypertension, Coronary disease, Diabetes, Heart Failure, Stroke, Dyslipidaemia, Hyperlipidaemia, Diabetes
Infectious diseases	HIV
Musculoskeletal diseases	Osteoporosis, osteoarthritis
Mental, behavioral or neurodevelopmental disorders	Schizophrenia, bipolar disorder, psychosis, depression, tobacco dependence

An overall composite score was defined for each study, as the proportion of adherent patients reported by any measure. If a study had more than one method of assessment, a mean adherence rate was calculated. The validation of this score has been previously described elsewhere (Tonin et al., 2018).

For optimal comparison and interpretation of the results, adherence interventions were classified into four categories: attitudinal, rewards, educational, and technical based on previous definitions (Roter et al., 1998; Demonceau et al., 2013; Sapkota et al., 2015). Usual care was defined as standard of care (SOC) for this analysis. Included studies could have a single component or combination of multiple components comprising their intervention. The definitions for the interventions can be found in Supplementary Material 3.

Risk of bias assessment was undertaken for all the primary studies included in the analysis. It was performed by two investigators using the Cochranene collaboration risk of bias Assessment tool (RoB) (Higgins et al., 2011).

### Data Analysis

A network meta-analysis using Bayesian framework was performed to compare the effectiveness of reported interventions on adherence rates of long-term interventions (with a follow-up of more than 10 months) across the condition groups previously described. This analysis was based on the Markov Chain Monte Carlo simulation. Transitivity analyses were performed by comparing population, interventions, and outcome definitions among the included studies. To analyse the multiple-arms studies a common heterogeneity parameter was considered and a conservative analysis of non-informative priors was conducted (Dias et al., 2010; Rucker et al., 2017).

Effect sizes measures were expressed as odds ratio (OR) with a 95% credibility interval (CrI). The goodness of fit of the model and consistency were assessed using the lowest residual deviance information criteria (DIC) between fixed and random-effect models tested. Convergence was attained based on visual inspection of Brooks-Gelman-Rubin plots and potential scale reduction factor-PSRF (1 < PSRF ≤ 1.05) (Dias et al., 2010; Higgins et al., 2012).

Ranking probabilities for each measure of adherence were calculated by using surface under the cumulative ranking analysis (SUCRA) to increase the estimate precision of the relative effect sizes of comparisons and to properly account for correlations between multi-arm trials (Mbuagbaw et al., 2017). SUCRA values can range from 0% (i.e., the intervention always ranks last) to 100% (it always ranks first).

Robustness of the network when having close-loops, was assessed via node-splitting analysis (*p* < 0.05 reveal significant inconsistencies in the network) (van Valkenhoef et al., 2016). Sensitivity analyses with the hypothetical removal or inclusion of the studies were conducted when discrepancies were identified in the network. All analyses were performed using software Addis version 1.17.6 (van Valkenhoef et al., 2013).

## Results

**Figure 1:**
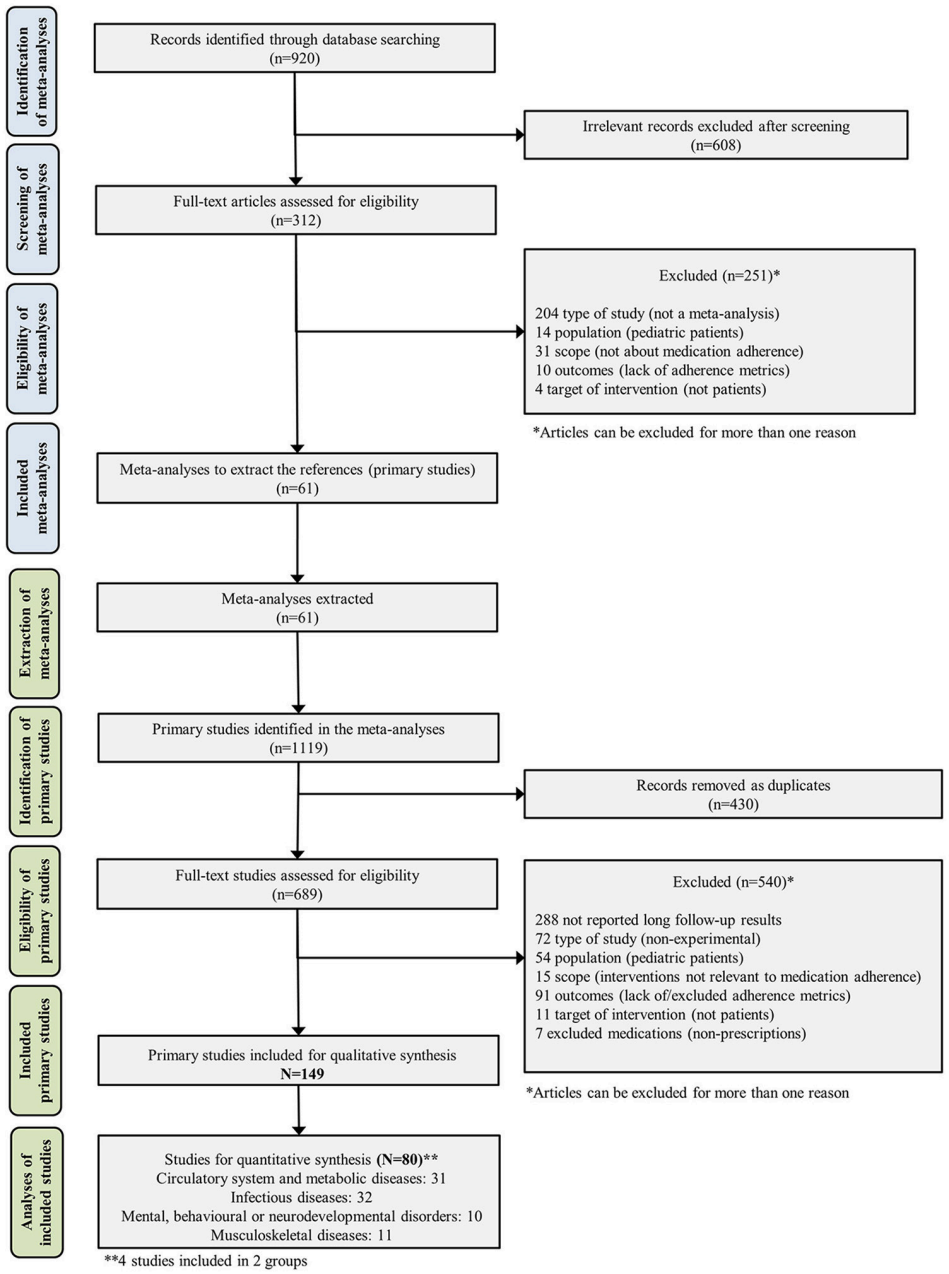
Flowchart of systematic review and network meta-analysis process.

A total of 920 records were identified and 61 meta-analyses, which included a median of 17.0 studies each [IQR 10.5–28.5; range 2–101], were finally selected for extraction of primary studies. From the selected meta-analyses, 1,119 primary studies were identified and 689 were assessed full-text for eligibility with 150 being included in the qualitative analysis and 80 in the network meta-analysis (Figure 1; and Supplementary Material 4). For those studies included in the qualitative synthesis, the publication years ranged between 1979 and 2016, with a median of 2007 (IQR 2006–2012). The number of studies per disease group was: 38 focused on infectious diseases (25.5%), 62 on circulatory system and metabolic diseases (41.6%), 13 on mental, behavioral or neurodevelopmental disorders (8.7%) and 14 on musculoskeletal diseases (9.4%). Five studies (3.4%) reported results in two groups of diseases and the remaining 16 studies (10.7%) corresponded to respiratory, digestive, transplant and undefined conditions. The only available articles classified into infectious diseases were focused on HIV (Human Immunodeficiency Virus).

Overall, 178,229 patients were included in the analyses, with the following distribution across disease groups: circulatory system and metabolic diseases (*n* = 59,959), infectious diseases (HIV) (*n* = 18,737), musculoskeletal diseases (*n* = 72,595) and mental, behavioral or neurodevelopmental disorders (*n* = 2,632). The average follow-up time was 14 months with the majority reporting a follow-up of 12 months (*n* = 115 studies). The most common interventions were educational (*n* = 49 studies, 28%), followed by educational + technical (*n* = 41, 23%), technical (*n* = 36, 20%), educational + attitudinal (*n* = 20, 11%), attitudinal (*n* = 21, 12%), educational + attitudinal + technical (*n* = 5, 3%) and only 3 studies (1.7%) containing the rewards component (rewards, rewards + technical, educational + attitudinal + rewards). In 134 studies (89.3%), standard care was used as a common comparator.

The risk of bias analysis resulted in a low risk on selective reporting (*n* = 146 studies, 98%) as all the papers reported the expected adherence outcomes. Around 20% of studies presented a high risk of bias for incomplete outcome data domain (*n* = 34) due to the lack of intention-to-treat analysis or missing data. Allocation concealment was classified as unclear risk of bias in most of the studies (*n* = 121, 81.2%). Additional information can be found in the Supplementary Material 5.

In the quantitative analysis, 80 studies were included, with 69 excluded due to the following reasons: (1) categorical medication adherence data not reported (*n* = 57), (2) same intervention category in all study arms (*n* = 3); and (3) not enough studies to be categorized and analyzed by disease group (*n* = 9).

**Figure 2:**
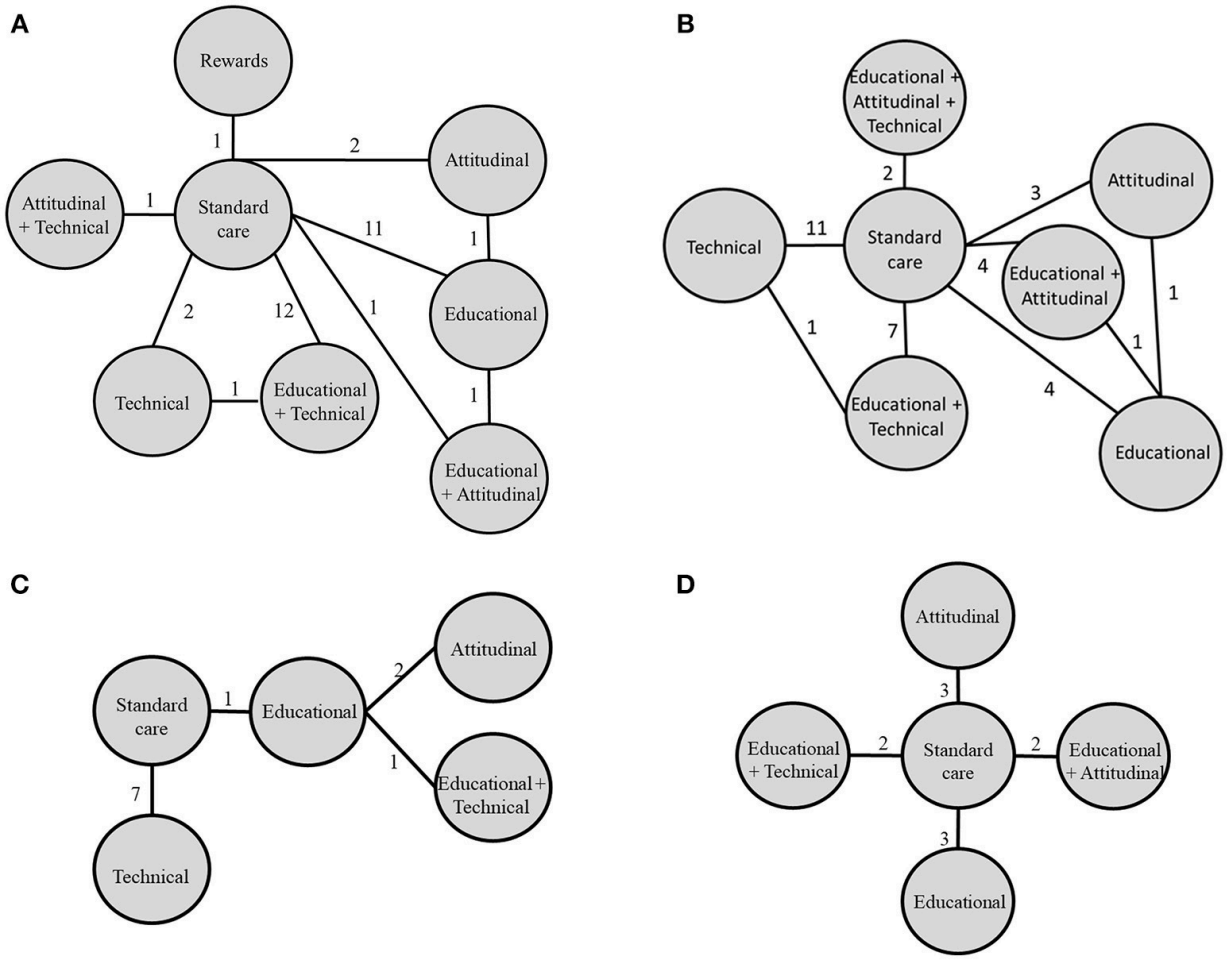
Networks diagrams of interventions on adherence across disease groups. Each line represents a direct comparison of interventions and the number of studies reporting that comparison is written on each line. From left to right: **(A)** Circulatory system and metabolic diseases, **(B)** Infectious diseases (HIV), **(C)** Musculoskeletal diseases, **(D)** Mental, behavioral or neurodevelopmental disorders.

Network meta-analyses were conducted per disease group (Figure 2), as described below. The list of included studies for each network meta-analysis can be found in Supplementary Material 6. Networks were found to be robust, with no significant inconsistency (Table 2 consistency analysis and Supplementary Material 7).

### Circulatory System and Metabolic Diseases

Thirty-one studies were included in this network, with seven different interventions being compared. Three studies assessed a combination of multiple intervention types, with a majority comparing educational + technical interventions (n = 12 studies) and educational interventions (*n* = 11) vs. SOC.

Educational + technical interventions were more effective in improving adherence when compared to SOC [OR: 0.44 (CrI: 0.26, 0.73)] (Table 2). In terms of ranking probabilities (SUCRA analysis), educational + technical interventions had the highest probability of being the best intervention at improving adherence in this disease group (79.6%). Technical interventions were ranked second (71.6%) and educational interventions third (55.9%). SOC ranked last (19.4%).

**Table 2:** Consistency analyses of comparisons on different group diseases based on composite score (A) Circulatory system and metabolic diseases; (B)Infectious diseases (HIV); (C) Mental, behavioral or neurodevelopmental disorders; (D) Musculoskeletal diseases.

**(A)**							
Attitudinal + Technical	0.84 (0.13, 4.88)	0.98 (0.11, 8.37)	0.96 (0.14, 6.67)	1.63 (0.34, 7.71)	1.13 (0.23, 5.34)	0.71 (0.15, 3.05)	1.47 (0.32, 6.52)
	Attitudinal	1.17 (0.18, 7.63)	1.15 (0.24, 5.35)	1.95 (0.65, 5.89)	1.35 (0.49, 3.81)	0.85 (0.32, 2.21)	1.76 (0.48, 6.53)
		Rewards	0.98 (0.13, 7.46)	1.65 (0.32, 9.14)	1.16 (0.22, 6.05)	0.72 (0.15, 3.52)	1.52 (0.24, 9.67)
		Educational + Attitudinal		1.69 (0.47, 6.61)	1.18 (0.35, 3.93)	0.74 (0.22, 2.46)	1.54 (0.35, 6.92)
				Educational + Technical	0.70 (0.33, 1.42)	**0.44 (0.26, 0.73)**	0.92 (0.34, 2.32)
					Educational	0.63 (0.38, 1.03)	1.31 (0.48, 3.60)
Circulatory system and Metabolic diseases						Standard Care	2.09 (0.86, 5.09)
							Technical
**(B)**							
Attitudinal	0.95 (0.33, 2.73)	1.08 (0.47, 2.60)	1.20 (0.58, 2.62)	0.91 (0.43, 1.82)	0.67 (0.37, 1.25)	1.09 (0.55, 2.25)	
	Educational + Attitudinal + Technical	1.14 (0.40, 3.40)	1.26 (0.48, 3.48)	0.96 (0.34, 2.69)	0.70 (0.30, 1.74)	1.15 (0.46, 3.09)	
		Educational + Attitudinal	1.12 (0.53, 2.28)	0.84 (0.37, 1.76)	0.62 (0.33, 1.12)	1.01 (0.51, 2.03)	
			Educational + Technical	0.76 (0.35, 1.48)	**0.56 (0.36, 0.84)**	0.91 (0.53, 1.57)	
				Educational	0.74 (0.43, 1.32)	1.20 (0.65, 2.49)	
Infectious diseases (HIV)					Standard Care	**1.63 (1.16, 2.38)**	
						Technical	
**(C)**							
Attitudinal	1.20 (0.21, 6.45)		0.45 (0.09, 2.33)		1.13 (0.25, 4.83)	0.45 (0.15, 1.29)	
	Educational + Attitudinal		0.38 (0.06, 2.21)		0.96 (0.18, 4.52)	0.37 (0.10, 1.37)	
			Educational + Technical		2.49 (0.50, 11.55)	0.98 (0.29, 3.50)	
Mental, behavioral or neurodevelopmental disorders					Educational	0.39 (0.15, 1.11)	
						Standard Care	
**(D)**							
Attitudinal	0.26 (0.05, 1.13)	0.80 (0.53, 1.36)	**0.30 (0.10, 0.86)**	0.47 (0.16, 1.41)			
	Educational + Technical	3.08 (0.77, 14.25)	1.14 (0.20, 6.59)	1.81 (0.32, 10.87)			
		Educational	**0.37 (0.14, 0.91)**	0.59 (0.21, 1.52)			
			Standard Care	**1.60 (1.26, 1.98)**			
Musculoskeletal diseases				Technical			

*Comparisons should be read left to right. The common cell between the intervention on the row and the one on the column represents the size effect, reported as OR (95% CrI). When comparing, values < 1 indicate the intervention in the row is more effective. Values >1 indicate the intervention in the column is more effective (e.g., for HIV, comparing Edu+Tec and SOC results on OR:0.56, lower than 1, indicating Edu+Tec is more effective than SOC). Statistically significant results are in bold*.

### Infectious Diseases: HIV

A total of 32 studies were included in this network with 6 different interventions. Three of these interventions were multicomponent. The majority of studies compared technical (*n* = 11) or educational + technical (*n* = 7) against SOC.

There were significant differences favoring educational + technical interventions [OR: 0.56 (0.36, 0.84)] and technical interventions [OR: 1.63 (1.16, 2.38)] compared to SOC (Table 2). SUCRA analysis showed educational + technical as the most probable to enhance adherence with a likelihood of 73.7%, followed by technical (63.2%) and educational + attitudinal (61.0%). Again, SOC ranked last (8.5%).

### Musculoskeletal Diseases

A total of 11 studies with 4 intervention combinations were analyzed in this network. The educational + technical interventions were used in 7 studies and were compared to SOC. Consistency analysis revealed statistical differences between attitudinal [OR: 0.30 (0.10, 0.86)], educational [OR: 0.37 (0.14, 0.91)] and technical [OR: 1.60 (1.26, 1.98)] interventions compared to SOC (Table 2).

Attitudinal interventions had the greatest probability of being the best option (92.3%) when compared to the other interventions. Educational (74.0%) and technical (48.3%) interventions ranked second and third, respectively. The lowest effect was for SOC (14.8%).

### Mental, Behavioral, or Neurodevelopmental Disorders Diseases

This network was comprised of 10 studies and compared 2 single component interventions, 2 combination interventions and standard care. Three studies assessed attitudinal interventions and three evaluated educational interventions. Two included educational + technical interventions and another two studies assessed educational + attitudinal interventions. All interventions were compared to SOC. No significant differences were found between types of interventions for this disease group (Table 2).

According to the SUCRA analysis, educational + attitudinal interventions ranked first (73.8%). Second and third rankings consisted of educational (72.5%) and attitudinal (65.3%) interventions respectively (See SUCRAS in Supplementary Material 8).

## Discussion

To the best of our knowledge, this is the first network meta-analysis assessing the comparative effectiveness of interventions aimed at improving medication adherence to chronic medications across different disease groups, with long-term follow-up periods. Differences in the effects of the interventions were found by disease groups, suggesting that adherence interventions should be adapted to the condition being targeted. There are numerous condition-related determinants affecting medication adherence (e.g., presence of symptoms, disease severity, clinical improvement, duration of the disease, psychiatric conditions) that require tailored and multifaceted approaches (Kardas et al., 2013).

Adherence interventions in circulatory system and metabolic diseases and infectious diseases (HIV) were significantly more effective when combining educational + technical components (with SUCRA values between 70 and 80%). Interventions involving educational components only (i.e., interventions providing information regarding the medication, disease state or importance of adherence with the aim of increasing a patient's knowledge or skills that facilitate adherence) are one of the most frequent strategies used in health care to change patient behavior (Sapkota et al., 2015). As hypothesized by the Information-Motivation-Strategy model (IMS) (DiMatteo et al., 2012), “patients are only capable of doing what they clearly understand,” emphasizing the importance of adequate patient information and knowledge to follow a treatment regimen (DiMatteo et al., 2012). However, the effectiveness of information provision and its effect on medication adherence can be affected by a range of healthcare team and system-related factors, such as poor patient-physician communication, patient's lack of trust, lack of shared decision making or poor follow-up amongst others (Kardas et al., 2013). Moreover, there is evidence a high proportion of patients are unable to remember the information provided during a medical consultation, highlighting that although essential, the provision of information as an isolated strategy can be insufficient to ensure medication adherence (Kravitz et al., 1993). Also suggested by the IMS model, patients can be non-adherent if they lack a strategy that allows them to follow their health care provider's recommendations (DiMatteo et al., 2012), as found especially evident in unintentional non-adherence (Horne et al., 2005). Patients must have the strategies and resources to be able to overcome practical barriers faced when attempting to follow their health care provider's recommendations (DiMatteo et al., 2012). Therefore, adding the use of technical components, that is interventions providing any gadget, instrument, or system that facilitate medication intake or increase convenience of the medication taking process, may increase medication adherence. These interventions often help patients adopt routines of medication taking when they have memory problems or have busy social lives that limit their ability to be adherent (Vervloet et al., 2012).

The results obtained for circulatory system and metabolic diseases and infectious diseases (HIV) are in agreement with previous literature reporting an increased effect when combining different interventions components (Kanters et al., 2017). A more specific analysis conducted in Africa revealed that adding educational and technical components to standard care could improve medication adherence (Mills et al., 2014). Other technical components such as regimen simplification, available for some of the medications used for HIV treatment, resulted in an increase on adherence as it reduces pill burden (Parienti et al., 2009; Nachega et al., 2014). There is also a reduction in treatment complexity and polypharmacy, important barriers preventing patients to adhere to their medications (Marcum and Gellad, 2012). Additionally, patients have to integrate doses into daily life, a process that may sometime represent shame or fear associated with the condition stigma. Minimizing this process may also reduce burden (Katz et al., 2013).

Attitudinal interventions were found to have the best effect to increase medication adherence in patients suffering from musculoskeletal diseases, with a SUCRA of 99.25% and were found to be significantly different to standard care. These findings indicate there is a strong effect from the use of behavior change theories on the improvement of medication adherence on these diseases. This might be due to a higher prevalence of intentional non-adherence (Horne and Weinman, 1999) in patients with these conditions. The Health Belief Model suggests that a health behavior can be influenced by perceived susceptibility, severity, benefits and barriers regarding a disease or condition (Glanz et al., 2015) and it has been suggested that effective relationships between physician and patients are necessary in order to help them to cope with medication non-adherence problems (DiMatteo et al., 2007). Therefore, behavior based theories that can be provided by physicians, such as motivational interviewing, can be used to improve adherence (Easthall et al., 2013). These often consist of focused skills to help the patient solve ambivalence and find solutions (Miller and Rollnick, 2012).

Consistency analysis did not show significant differences between the effectiveness of different interventions for patients with mental, behavioral or neurodevelopmental disorders. A reason for these results may be because adherence is complex and dynamic and requires accurate assessment of practical and motivational barriers due to external factors associated to the condition itself (Chapman and Horne, 2013). However, the combination of educational + attitudinal components presented higher SUCRA values (around 75%). These results are congruent with previous research that showed that incorporating attitudinal interventions, such as psychoeducation, are an effective strategy to increase on medication adherence in patients with mental disorders (Bond and Anderson, 2015; Hartung et al., 2017). Attitudes and beliefs about the need to take medications can be moderated by the condition itself, such as dependence, the feeling of medications controlling their attitudes, or impact of medicines on daily routines (Chakrabarti, 2016).

Rewards type interventions, interventions that provide incentives, awards or penalties to facilitate medication adherence, were evaluated only for one study and for one disease group (circulatory system and metabolic diseases) with no significant long-term effect compared to other interventions or SOC. The intervention was focused on full payment coverage of medications (Choudhry et al., 2011). Usually, the application of this type of intervention requires modifications on health policies (e.g., coverage of medications) and involves ethical concerns of providing incentives to patients (Noordraven et al., 2017).

The limitations of this study include the categorization of the interventions into four major groups to perform the network meta-analyses. We acknowledge that a different categorization system may lead to some different results. However, this classification system, which was developed based on three previously used classifications, allowed us to have a clearer understanding of the interventions (Roter et al., 1998; Demonceau et al., 2013; Sapkota et al., 2015). The use of different classification systems for the clinical conditions may also lead to different results. We used the standard groups proposed by the International Classification of Diseases from the World Health Organization. Some other important conditions groups such as respiratory (e.g., asthma, COPD) could not be compared because of the lack of studies reporting long-term categorical outcomes on adherence. Results on adherence were focused only on implementation, one of the components of the current adherence definition proposed by ABC Project Team (Vrijens et al., 2012) as there were not enough studies reporting initiation or discontinuation adherence that could be analyzed. We used a previously validated composite measure of adherence to consider in one single model different individual measures and provide a broad evaluation of the effectiveness of complex interventions. The use of other measures can produce slightly different results.

## Conclusion

Educational and technical interventions seem to be more effective on the long-term management of medication adherence in patients with HIV, circulatory system and metabolic diseases, compared to attitudinal interventions that presented a superior effect on mental, behavioral or neurodevelopmental disorders and musculoskeletal diseases. Multicomponent interventions are more effective at enhancing medication adherence in three of the four disease groups. Further analyses assessing the impact of these interventions on clinical outcomes are needed to support the translation of these results to daily practice. The use of network meta-analysis was valuable for comparing interventions aimed to improve medication adherence across chronic diseases in long-term follow-up periods.

## Author Contributions

VG-C, SB, FF-L, AT-R, EW, and FT contributed to the design of the study. EW and AT-R organized the database. FT and FF-L performed the data analysis. AT-R wrote the first draft of this manuscript. All authors contributed to manuscript revision, read and approved the submitted version.

## Conflict of Interest Statement

The authors declare that the research was conducted in the absence of any commercial or financial relationships that could be construed as a potential conflict of interest.
