# Supplementary material for: Comparison of Interventions to Improve Long-Term Medication Adherence Across Different Clinical Conditions: A Systematic Review With Network Meta-Analysis
Source: Front Pharmacol. 2018 Dec 24;9:1454. doi: 10.3389/fphar.2018.01454 (PMC6311651; doi:10.3389/fphar.2018.01454)
Supplement: Supplementary file 1 [file Data_Sheet_1.docx]

Supplementary Material

Comparison of interventions to improve long-term medication adherence across different clinical conditions: a systematic review with network meta-analysis

**Andrea Torres-Robles, Elyssa Wiecek, Fernanda S. Tonin, Shalom I. Benrimoj, Fernando Fernandez-Llimos, Victoria Garcia-Cardenas***

*** Correspondence:** Corresponding Author: Victoria.garciacardenas@uts.edu.au

**Supplementary Material**

1. Complete search strategy
2. Complete inclusion and exclusion criteria
3. Category definitions
4. Complete references
   1. Included meta-analyses
   2. Included primary studies
   3. Excluded primary studies
5. Risk of bias assessment
6. Studies included in the network meta-analysis
7. Node-splitting analyses
8. SUCRA analyses
9. **Complete search strategy**

| **PubMed** | **#1** (“drug therapy”[Mesh Terms] OR “medication[Title/Abstract]) AND (“patient compliance”[Mesh Terms] OR “medication adherence”[Mesh Terms] OR “medication adherence”[Title/Abstract])  **#2** “systematic review”[Title/Abstract] OR “meta-analysis”[Publication type] OR “meta-analysis”[Title/Abstract]  **#1 AND #2** |
| --- | --- |

1. **Complete inclusion and exclusion criteria**

**Meta-analyses**

We are looking for meta-analyses focused on medication adherence interventions with adherence outcomes including: pill count, refill data, self-report, or electronic monitoring.

Excluded

1. Meta-Analysis not performed or studies in meta-analysis not listed
2. Only paediatric studies included (<18 years)
3. No medication adherence intervention studies included (i.e. only medication efficacy studies)
4. No medication adherence data reported
5. No outcomes of adherence reported including: pill count, refill data, self-report, and electronic monitoring (i.e. clinical outcomes only) or only includes outcomes that are not assessable (i.e. drug levels/depot medications/etc.)
6. Only provider/healthcare professional targeted interventions and outcomes

**Primary studies**

We are looking for experimental studies with interventions aimed at adult patients on prescription medications with adherence outcomes including: pill count, refill data, self-report, or electronic monitoring.

Exclusion criteria

1. Publications not subject to peer-review, conference posters/abstracts, dissertations, or unpublished data sets
2. Expert opinion pieces, economic analyses, single case reports, cross-sectional studies (i.e. retrospective data on once vs twice daily), medication efficacy studies with no adherence intervention, or cohort studies
3. Children < 18 years included in study or studies aimed at physicians/healthcare professionals
4. Over-the-counter medications, depot medications, vaccines, any medication without instructions or with “as needed” instructions where the patient decides the dose
5. Studies only measuring clinical outcomes, drug levels, undefined adherence outcomes, or initiation or discontinuation adherence
6. Treatment follow-up less than 10 months.
7. **Category definitions**

| **Category** | **Definition** |
| --- | --- |
| Educational | Interventions providing information regarding the medication, disease state or importance of adherence, in any form (e.g. written, oral, in group, by telephone), to a patient with the aim of increasing a patient’s knowledge or skills that facilitate adherence. |
| Attitudinal | Interventions aiming to modify behavioral intention based on modifying patient’s attitudes, beliefs or subjective norm related to their disease state or medication (e.g. motivational interviewing, cognitive behavioral therapy, etc.), delivered in any form (e.g. written, oral, in group, by telephone). |
| Technical | Interventions providing any gadget, instrument, or system that facilitate the medication intake or increase convenience of the medication taking process, such as reminders, regime simplifications, telephone follow-ups, direction observation therapy, self-monitoring, cue-dose training, electronic monitoring feedback etc. |
| Rewards | Interventions that provide incentives, awards or penalties to facilitate medication adherence. |

1. **Complete references**
   1. **Included meta-analyses**

1. Bangalore S, Kamalakkannan G, Parkar S, Messerli FH. Fixed-dose combinations improve medication compliance: a meta-analysis. Am J Med. 2007;120(8):713-9.

2. Burudpakdee C, Khan ZM, Gala S, Nanavaty M, Kaura S. Impact of patient programs on adherence and persistence in inflammatory and immunologic diseases: a meta-analysis. Patient Prefer Adherence. 2015;9:435-48.

3. Caldeira D, Vaz-Carneiro A, Costa J. The impact of dosing frequency on medication adherence in chronic cardiovascular disease: systematic review and meta-analysis. Rev Port Cardiol. 2014;33(7-8):431-7.

4. Chase JA, Bogener JL, Ruppar TM, Conn VS. The Effectiveness of Medication Adherence Interventions Among Patients With Coronary Artery Disease: A Meta-analysis. J Cardiovasc Nurs. 2016;31(4):357-66.

5. Conn VS, Enriquez M, Ruppar TM, Chan KC. Cultural relevance in medication adherence interventions with underrepresented adults: systematic review and meta-analysis of outcomes. Prev Med. 2014;69:239-47.

6. Conn VS, Hafdahl AR, Cooper PS, Ruppar TM, Mehr DR, Russell CL. Interventions to improve medication adherence among older adults: meta-analysis of adherence outcomes among randomized controlled trials. Gerontologist. 2009;49(4):447-62.

7. Conn VS, Ruppar TM, Chan KC, Dunbar-Jacob J, Pepper GA, De Geest S. Packaging interventions to increase medication adherence: systematic review and meta-analysis. Curr Med Res Opin. 2015;31(1):145-60.

8. Conn VS, Ruppar TM, Chase JA, Enriquez M, Cooper PS. Interventions to Improve Medication Adherence in Hypertensive Patients: Systematic Review and Meta-analysis. Curr Hypertens Rep. 2015;17(12):94.

9. Conn VS, Ruppar TM, Enriquez M, Cooper P. Medication adherence interventions that target subjects with adherence problems: Systematic review and meta-analysis. Res Social Adm Pharm. 2016;12(2):218-46.

10. de Bruin M, Viechtbauer W, Schaalma HP, Kok G, Abraham C, Hospers HJ. Standard care impact on effects of highly active antiretroviral therapy adherence interventions: A meta-analysis of randomized controlled trials. Arch Intern Med. 2010;170(3):240-50.

11. De Simoni A, Hardeman W, Mant J, Farmer AJ, Kinmonth AL. Trials to improve blood pressure through adherence to antihypertensives in stroke/TIA: systematic review and meta-analysis. J Am Heart Assoc. 2013;2(4):e000251.

12. Demonceau J, Ruppar T, Kristanto P, Hughes DA, Fargher E, Kardas P, et al. Identification and assessment of adherence-enhancing interventions in studies assessing medication adherence through electronically compiled drug dosing histories: a systematic literature review and meta-analysis. Drugs. 2013;73(6):545-62.

13. Easthall C, Song F, Bhattacharya D. A meta-analysis of cognitive-based behaviour change techniques as interventions to improve medication adherence. BMJ Open. 2013;3(8).

14. Falagas ME, Karagiannis AK, Nakouti T, Tansarli GS. Compliance with once-daily versus twice or thrice-daily administration of antibiotic regimens: a meta-analysis of randomized controlled trials. PLoS One. 2015;10(1):e0116207.

15. Fenerty SD, West C, Davis SA, Kaplan SG, Feldman SR. The effect of reminder systems on patients' adherence to treatment. Patient Prefer Adherence. 2012;6:127-35.

16. Finitsis DJ, Pellowski JA, Johnson BT. Text message intervention designs to promote adherence to antiretroviral therapy (ART): a meta-analysis of randomized controlled trials. PLoS One. 2014;9(2):e88166.

17. Fletcher BR, Hartmann-Boyce J, Hinton L, McManus RJ. The Effect of Self-Monitoring of Blood Pressure on Medication Adherence and Lifestyle Factors: A Systematic Review and Meta-Analysis. Am J Hypertens. 2015;28(10):1209-21.

18. Gray R, Bressington D, Ivanecka A, Hardy S, Jones M, Schulz M, et al. Is adherence therapy an effective adjunct treatment for patients with schizophrenia spectrum disorders? A systematic review and meta-analysis. BMC Psychiatry. 2016;16:90.

19. Hart JE, Jeon CY, Ivers LC, Behforouz HL, Caldas A, Drobac PC, et al. Effect of directly observed therapy for highly active antiretroviral therapy on virologic, immunologic, and adherence outcomes: a meta-analysis and systematic review. J Acquir Immune Defic Syndr. 2010;54(2):167-79.

20. Heneghan CJ, Glasziou P, Perera R. Reminder packaging for improving adherence to self-administered long-term medications. Cochrane Database Syst Rev. 2006(1):CD005025.

21. Hollands GJ, McDermott MS, Lindson-Hawley N, Vogt F, Farley A, Aveyard P. Interventions to increase adherence to medications for tobacco dependence. Cochrane Database Syst Rev. 2015(2):CD009164.

22. Horvath T, Azman H, Kennedy GE, Rutherford GW. Mobile phone text messaging for promoting adherence to antiretroviral therapy in patients with HIV infection. Cochrane Database Syst Rev. 2012(3):CD009756.

23. Iglay K, Cao X, Mavros P, Joshi K, Yu S, Tunceli K. Systematic Literature Review and Meta-analysis of Medication Adherence With Once-weekly Versus Once-daily Therapy. Clin Ther. 2015;37(8):1813-21 e1.

24. Iskedjian M, Einarson TR, MacKeigan LD, Shear N, Addis A, Mittmann N, et al. Relationship between daily dose frequency and adherence to antihypertensive pharmacotherapy: evidence from a meta-analysis. Clin Ther. 2002;24(2):302-16.

25. Kanters S, Park JJ, Chan K, Ford N, Forrest J, Thorlund K, et al. Use of peers to improve adherence to antiretroviral therapy: a global network meta-analysis. J Int AIDS Soc. 2016;19(1):21141.

26. Kanters S, Park JJ, Chan K, Socias ME, Ford N, Forrest JI, et al. Interventions to improve adherence to antiretroviral therapy: a systematic review and network meta-analysis. Lancet HIV. 2017;4(1):e31-e40.

27. Kassavou A, Sutton S. Automated telecommunication interventions to promote adherence to cardio-metabolic medications: meta-analysis of effectiveness and meta-regression of behaviour change techniques. Health Psychol Rev. 2018;12(1):25-42.

28. Kauppi K, Valimaki M, Hatonen HM, Kuosmanen LM, Warwick-Smith K, Adams CE. Information and communication technology based prompting for treatment compliance for people with serious mental illness. Cochrane Database Syst Rev. 2014(6):CD009960.

29. Langebeek N, Nieuwkerk P. Electronic medication monitoring-informed counseling to improve adherence to combination anti-retroviral therapy and virologic treatment outcomes: a meta-analysis. Front Public Health. 2015;3:139.

30. MacDonald L, Chapman S, Syrett M, Bowskill R, Horne R. Improving medication adherence in bipolar disorder: A systematic review and meta-analysis of 30 years of intervention trials. J Affect Disord. 2016;194:202-21.

31. Mahtani KR, Heneghan CJ, Glasziou PP, Perera R. Reminder packaging for improving adherence to self-administered long-term medications. Cochrane Database Syst Rev. 2011(9):CD005025.

32. Manias E, Williams A. Medication adherence in people of culturally and linguistically diverse backgrounds: a meta-analysis. Ann Pharmacother. 2010;44(6):964-82.

33. Miller L, Schuz B, Walters J, Walters EH. Mobile Technology Interventions for Asthma Self-Management: Systematic Review and Meta-Analysis. JMIR Mhealth Uhealth. 2017;5(5):e57.

34. Morrissey EC, Durand H, Nieuwlaat R, Navarro T, Haynes RB, Walsh JC, et al. Effectiveness and content analysis of interventions to enhance medication adherence and blood pressure control in hypertension: A systematic review and meta-analysis. Psychol Health. 2017;32(10):1195-232.

35. Mutasa-Apollo T, Ford N, Wiens M, Socias ME, Negussie E, Wu P, et al. Effect of frequency of clinic visits and medication pick-up on antiretroviral treatment outcomes: a systematic literature review and meta-analysis. J Int AIDS Soc. 2017;20(Suppl 4):21647.

36. Normansell R, Kew KM, Stovold E. Interventions to improve adherence to inhaled steroids for asthma. Cochrane Database Syst Rev. 2017;4:CD012226.

37. Palacio A, Garay D, Langer B, Taylor J, Wood BA, Tamariz L. Motivational Interviewing Improves Medication Adherence: a Systematic Review and Meta-analysis. J Gen Intern Med. 2016;31(8):929-40.

38. Parienti JJ, Bangsberg DR, Verdon R, Gardner EM. Better adherence with once-daily antiretroviral regimens: a meta-analysis. Clin Infect Dis. 2009;48(4):484-8.

39. Peterson AM, Takiya L, Finley R. Meta-analysis of trials of interventions to improve medication adherence. Am J Health Syst Pharm. 2003;60(7):657-65.

40. Petry NM, Rash CJ, Byrne S, Ashraf S, White WB. Financial reinforcers for improving medication adherence: findings from a meta-analysis. Am J Med. 2012;125(9):888-96.

41. Rash JA, Campbell DJ, Tonelli M, Campbell TS. A systematic review of interventions to improve adherence to statin medication: What do we know about what works? Prev Med. 2016;90:155-69.

42. Readdean KC, Heuer AJ, Scott Parrott J. Effect of pharmacist intervention on improving antidepressant medication adherence and depression symptomology: A systematic review and meta-analysis. Res Social Adm Pharm. 2017.

43. Rocha BS, Silveira MP, Moraes CG, Kuchenbecker RS, Dal-Pizzol TS. Pharmaceutical interventions in antiretroviral therapy: systematic review and meta-analysis of randomized clinical trials. J Clin Pharm Ther. 2015;40(3):251-8.

44. Rubio-Valera M, Serrano-Blanco A, Magdalena-Belio J, Fernandez A, Garcia-Campayo J, Pujol MM, et al. Effectiveness of pharmacist care in the improvement of adherence to antidepressants: a systematic review and meta-analysis. Ann Pharmacother. 2011;45(1):39-48.

45. Ruppar TM, Delgado JM, Temple J. Medication adherence interventions for heart failure patients: A meta-analysis. Eur J Cardiovasc Nurs. 2015;14(5):395-404.

46. Ruppar TM, Dunbar-Jacob JM, Mehr DR, Lewis L, Conn VS. Medication adherence interventions among hypertensive black adults: a systematic review and meta-analysis. J Hypertens. 2017;35(6):1145-54.

47. Sakakibara BM, Kim AJ, Eng JJ. A Systematic Review and Meta-Analysis on Self-Management for Improving Risk Factor Control in Stroke Patients. Int J Behav Med. 2017;24(1):42-53.

48. Santo K, Kirkendall S, Laba TL, Thakkar J, Webster R, Chalmers J, et al. Interventions to improve medication adherence in coronary disease patients: A systematic review and meta-analysis of randomised controlled trials. Eur J Prev Cardiol. 2016;23(10):1065-76.

49. Simoni JM, Pearson CR, Pantalone DW, Marks G, Crepaz N. Efficacy of interventions in improving highly active antiretroviral therapy adherence and HIV-1 RNA viral load. A meta-analytic review of randomized controlled trials. J Acquir Immune Defic Syndr. 2006;43 Suppl 1:S23-35.

50. Sin NL, DiMatteo MR. Depression treatment enhances adherence to antiretroviral therapy: a meta-analysis. Ann Behav Med. 2014;47(3):259-69.

51. Takiya LN, Peterson AM, Finley RS. Meta-analysis of interventions for medication adherence to antihypertensives. Ann Pharmacother. 2004;38(10):1617-24.

52. Tao D, Xie L, Wang T, Wang T. A meta-analysis of the use of electronic reminders for patient adherence to medication in chronic disease care. J Telemed Telecare. 2015;21(1):3-13.

53. Thakkar J, Kurup R, Laba TL, Santo K, Thiagalingam A, Rodgers A, et al. Mobile Telephone Text Messaging for Medication Adherence in Chronic Disease: A Meta-analysis. JAMA Intern Med. 2016;176(3):340-9.

54. Van Camp YP, Van Rompaey B, Elseviers MM. Nurse-led interventions to enhance adherence to chronic medication: systematic review and meta-analysis of randomised controlled trials. Eur J Clin Pharmacol. 2013;69(4):761-70.

55. van Driel ML, Morledge MD, Ulep R, Shaffer JP, Davies P, Deichmann R. Interventions to improve adherence to lipid-lowering medication. Cochrane Database Syst Rev. 2016;12:CD004371.

56. van Galen KA, Nellen JF, Nieuwkerk PT. The Effect on Treatment Adherence of Administering Drugs as Fixed-Dose Combinations versus as Separate Pills: Systematic Review and Meta-Analysis. AIDS Res Treat. 2014;2014:967073.

57. Vignon Zomahoun HT, de Bruin M, Guillaumie L, Moisan J, Gregoire JP, Perez N, et al. Effectiveness and Content Analysis of Interventions to Enhance Oral Antidiabetic Drug Adherence in Adults with Type 2 Diabetes: Systematic Review and Meta-Analysis. Value Health. 2015;18(4):530-40.

58. Wald DS, Butt S, Bestwick JP. One-way versus two-way text messaging on improving medication adherence: meta-analysis of randomized trials. Am J Med. 2015;128(10):1139 e1-5.

59. Zhu Y, Zhou Y, Zhang L, Zhang J, Lin J. Efficacy of interventions for adherence to the immunosuppressive therapy in kidney transplant recipients: a meta-analysis and systematic review. J Investig Med. 2017;65(7):1049-56.

60. Zomahoun HTV, Guenette L, Gregoire JP, Lauzier S, Lawani AM, Ferdynus C, et al. Effectiveness of motivational interviewing interventions on medication adherence in adults with chronic diseases: a systematic review and meta-analysis. Int J Epidemiol. 2017;46(2):589-602.

61. Zou H, Li Z, Nolan MT, Arthur D, Wang H, Hu L. Self-management education interventions for persons with schizophrenia: a meta-analysis. Int J Ment Health Nurs. 2013;22(3):256-71.

- 1. **References from all included original studies in qualitative review**

1. Amado Guirado E, Pujol Ribera E, Pacheco Huergo V, Borras JM. Knowledge and adherence to antihypertensive therapy in primary care: results of a randomized trial. Gac Sanit. 2011;25(1):62-7.
2. Antoni MH, Carrico AW, Duran RE, Spitzer S, Penedo F, Ironson G, et al. Randomized clinical trial of cognitive behavioral stress management on human immunodeficiency virus viral load in gay men treated with highly active antiretroviral therapy. Psychosom Med. 2006;68(1):143-51.
3. Antonicelli R, Mazzanti I, Abbatecola AM, Parati G. Impact of home patient telemonitoring on use of beta-blockers in congestive heart failure. Drugs Aging. 2010;27(10):801-5.
4. Bailey WC, Richards JM, Jr., Brooks CM, Soong SJ, Windsor RA, Manzella BA. A randomized trial to improve self-management practices of adults with asthma. Arch Intern Med. 1990;150(8):1664-8.
5. Ball JR, Mitchell PB, Corry JC, Skillecorn A, Smith M, Malhi GS. A randomized controlled trial of cognitive therapy for bipolar disorder: focus on long-term change. J Clin Psychiatry. 2006;67(2):277-86.
6. Begley S, Livingstone C, Hodges N, Williamson V. Impact of domiciliary pharmacy visits on medication management in an elderly population. International Journal of Pharmacy Practice. 1997;5(3):111-21.
7. Berger S, Schad T, von Wyl V, Ehlert U, Zellweger C, Furrer H, et al. Effects of cognitive behavioral stress management on HIV-1 RNA, CD4 cell counts and psychosocial parameters of HIV-infected persons. AIDS. 2008;22(6):767-75.
8. Bisharat B, Hafi L, Baron-Epel O, Armaly Z, Bowirrat A. Pharmacist counseling to cardiac patients in Israel prior to discharge from hospital contribute to increasing patient's medication adherence closing gaps and improving outcomes. J Transl Med. 2012;10:34.
9. Bobrow K, Farmer AJ, Springer D, Shanyinde M, Yu LM, Brennan T, et al. Mobile Phone Text Messages to Support Treatment Adherence in Adults With High Blood Pressure (SMS-Text Adherence Support [StAR]): A Single-Blind, Randomized Trial. Circulation. 2016;133(6):592-600.
10. The MEDMAN study: a randomized controlled trial of community pharmacy-led medicines management for patients with coronary heart disease. Family practice. 2007;24(2):189-200.
11. Boyle BA, Jayaweera D, Witt MD, Grimm K, Maa JF, Seekins DW. Randomization to once-daily stavudine extended release/lamivudine/efavirenz versus a more frequent regimen improves adherence while maintaining viral suppression. HIV Clin Trials. 2008;9(3):164-76.
12. Brankin E, Walker M, Lynch N, Aspray T, Lis Y, Cowell W. The impact of dosing frequency on compliance and persistence with bisphosphonates among postmenopausal women in the UK: evidence from three databases. Curr Med Res Opin. 2006;22(7):1249-56.
13. Broekhuizen K, van Poppel MN, Koppes LL, Kindt I, Brug J, van Mechelen W. Can multiple lifestyle behaviours be improved in people with familial hypercholesterolemia? Results of a parallel randomised controlled trial. PLoS One. 2012;7(12):e50032.
14. Capoccia KL, Boudreau DM, Blough DK, Ellsworth AJ, Clark DR, Stevens NG, et al. Randomized trial of pharmacist interventions to improve depression care and outcomes in primary care. Am J Health Syst Pharm. 2004;61(4):364-72.
15. Chan SS, Leung DY, Abdullah AS, Lo SS, Yip AW, Kok WM, et al. Smoking-cessation and adherence intervention among Chinese patients with erectile dysfunction. Am J Prev Med. 2010;39(3):251-8.
16. Chan SS, Leung DY, Abdullah AS, Wong VT, Hedley AJ, Lam TH. A randomized controlled trial of a smoking reduction plus nicotine replacement therapy intervention for smokers not willing to quit smoking. Addiction. 2011;106(6):1155-63.
17. Chang LW, Kagaayi J, Nakigozi G, Ssempijja V, Packer AH, Serwadda D, et al. Effect of peer health workers on AIDS care in Rakai, Uganda: a cluster-randomized trial. PLoS One. 2010;5(6):e10923.
18. Chisholm MA, Mulloy LL, Jagadeesan M, DiPiro JT. Impact of clinical pharmacy services on renal transplant patients' compliance with immunosuppressive medications. Clin Transplant. 2001;15(5):330-6.
19. Choudhry NK, Avorn J, Glynn RJ, Antman EM, Schneeweiss S, Toscano M, et al. Full coverage for preventive medications after myocardial infarction. N Engl J Med. 2011;365(22):2088-97.
20. Clowes JA, Peel NF, Eastell R. The impact of monitoring on adherence and persistence with antiresorptive treatment for postmenopausal osteoporosis: a randomized controlled trial. J Clin Endocrinol Metab. 2004;89(3):1117-23.
21. Collier AC, Ribaudo H, Mukherjee AL, Feinberg J, Fischl MA, Chesney M. A randomized study of serial telephone call support to increase adherence and thereby improve virologic outcome in persons initiating antiretroviral therapy. J Infect Dis. 2005;192(8):1398-406.
22. Cramer JA, Amonkar MM, Hebborn A, Altman R. Compliance and persistence with bisphosphonate dosing regimens among women with postmenopausal osteoporosis. Curr Med Res Opin. 2005;21(9):1453-60.
23. Cramer JA, Lynch NO, Gaudin AF, Walker M, Cowell W. The effect of dosing frequency on compliance and persistence with bisphosphonate therapy in postmenopausal women: a comparison of studies in the United States, the United Kingdom, and France. Clin Ther. 2006;28(10):1686-94.
24. Delmas PD, Vrijens B, Eastell R, Roux C, Pols HA, Ringe JD, et al. Effect of monitoring bone turnover markers on persistence with risedronate treatment of postmenopausal osteoporosis. J Clin Endocrinol Metab. 2007;92(4):1296-304.
25. Derose SF, Green K, Marrett E, Tunceli K, Cheetham TC, Chiu VY, et al. Automated outreach to increase primary adherence to cholesterol-lowering medications. JAMA Intern Med. 2013;173(1):38-43.
26. DiIorio C, McCarty F, Resnicow K, McDonnell Holstad M, Soet J, Yeager K, et al. Using motivational interviewing to promote adherence to antiretroviral medications: a randomized controlled study. AIDS Care. 2008;20(3):273-83.
27. D'Souza R, Piskulic D, Sundram S. A brief dyadic group based psychoeducation program improves relapse rates in recently remitted bipolar disorder: a pilot randomised controlled trial. J Affect Disord. 2010;120(1-3):272-6.
28. Edworthy SM, Baptie B, Galvin D, Brant RF, Churchill-Smith T, Manyari D, et al. Effects of an enhanced secondary prevention program for patients with heart disease: a prospective randomized trial. Can J Cardiol. 2007;23(13):1066-72.
29. Eron JJ, Feinberg J, Kessler HA, Horowitz HW, Witt MD, Carpio FF, et al. Once-daily versus twice-daily lopinavir/ritonavir in antiretroviral-naive HIV-positive patients: a 48-week randomized clinical trial. J Infect Dis. 2004;189(2):265-72.
30. Eussen SR, van der Elst ME, Klungel OH, Rompelberg CJ, Garssen J, Oosterveld MH, et al. A pharmaceutical care program to improve adherence to statin therapy: a randomized controlled trial. Ann Pharmacother. 2010;44(12):1905-13.
31. Falces C, Lopez-Cabezas C, Andrea R, Arnau A, Ylla M, Sadurni J. [An educative intervention to improve treatment compliance and to prevent readmissions of elderly patients with heart failure]. Med Clin (Barc). 2008;131(12):452-6.
32. Farmer KC, Jacobs EW, Phillips CR. Long-term patient compliance with prescribed regimens of calcium channel blockers. Clin Ther. 1994;16(2):316-26; discussion 271-2.
33. Gallant JE, DeJesus E, Arribas JR, Pozniak AL, Gazzard B, Campo RE, et al. Tenofovir DF, emtricitabine, and efavirenz vs. zidovudine, lamivudine, and efavirenz for HIV. N Engl J Med. 2006;354(3):251-60.
34. Gamble J, Stevenson M, Heaney LG. A study of a multi-level intervention to improve non-adherence in difficult to control asthma. Respir Med. 2011;105(9):1308-15.
35. Glanz K, Beck AD, Bundy L, Primo S, Lynn MJ, Cleveland J, et al. Impact of a health communication intervention to improve glaucoma treatment adherence. Results of the interactive study to increase glaucoma adherence to treatment trial. Arch Ophthalmol. 2012;130(10):1252-8.
36. Goggin K, Gerkovich MM, Williams KB, Banderas JW, Catley D, Berkley-Patton J, et al. A randomized controlled trial examining the efficacy of motivational counseling with observed therapy for antiretroviral therapy adherence. AIDS Behav. 2013;17(6):1992-2001.
37. Goswami NJ, Dekoven M, Kuznik A, Mardekian J, Krukas MR, Liu LZ, et al. Impact of an integrated intervention program on atorvastatin adherence: a randomized controlled trial. Int J Gen Med. 2013;6:647-55.
38. Goujard C, Bernard N, Sohier N, Peyramond D, Lancon F, Chwalow J, et al. Impact of a patient education program on adherence to HIV medication: a randomized clinical trial. J Acquir Immune Defic Syndr. 2003;34(2):191-4.
39. Granger BB, Ekman I, Hernandez AF, Sawyer T, Bowers MT, DeWald TA, et al. Results of the Chronic Heart Failure Intervention to Improve MEdication Adherence study: A randomized intervention in high-risk patients. Am Heart J. 2015;169(4):539-48.
40. Gray R, Leese M, Bindman J, Becker T, Burti L, David A, et al. Adherence therapy for people with schizophrenia. European multicentre randomised controlled trial. Br J Psychiatry. 2006;189:508-14.
41. Gross R, Bellamy SL, Chapman J, Han X, O'Duor J, Palmer SC, et al. Managed problem solving for antiretroviral therapy adherence: a randomized trial. JAMA Intern Med. 2013;173(4):300-6.
42. Grymonpre RE, Williamson DA, Montgomery PR. Impact of a pharmaceutical care model for non-institutionalised elderly: results of a randomised, controlled trial. International Journal of Pharmacy Practice. 2001;9(4):235-41.
43. Amado Guirado E, Pujol Ribera E, Pacheco Huergo V, Borras JM. Knowledge and adherence to antihypertensive therapy in primary care: results of a randomized trial. Gaceta Sanitaria. 2011;25(1):62-7.
44. Gujral G, Winckel K, Nissen LM, Cottrell WN. Impact of community pharmacist intervention discussing patients' beliefs to improve medication adherence. Int J Clin Pharm. 2014;36(5):1048-58.
45. Gwadry-Sridhar FH, Arnold JM, Zhang Y, Brown JE, Marchiori G, Guyatt G. Pilot study to determine the impact of a multidisciplinary educational intervention in patients hospitalized with heart failure. Am Heart J. 2005;150(5):982.
46. Hawkins DW, Fiedler FP, Douglas HL, Eschbach RC. Evaluation of a clinical pharmacist in caring for hypertensive and diabetic patients. Am J Hosp Pharm. 1979;36(10):1321-5.
47. Hirsch JD, Gonzales M, Rosenquist A, Miller TA, Gilmer TP, Best BM. Antiretroviral therapy adherence, medication use, and health care costs during 3 years of a community pharmacy medication therapy management program for Medi-Cal beneficiaries with HIV/AIDS. J Manag Care Pharm. 2011;17(3):213-23.
48. Hirsch JD, Rosenquist A, Best BM, Miller TA, Gilmer TP. Evaluation of the first year of a pilot program in community pharmacy: HIV/AIDS medication therapy management for Medi-Cal beneficiaries. J Manag Care Pharm. 2009;15(1):32-41.
49. Ho PM, Lambert-Kerzner A, Carey EP, Fahdi IE, Bryson CL, Melnyk SD, et al. Multifaceted intervention to improve medication adherence and secondary prevention measures after acute coronary syndrome hospital discharge: a randomized clinical trial. JAMA Intern Med. 2014;174(2):186-93.
50. Homer D, Nightingale P, Jobanputra P. Providing patients with information about disease-modifying anti-rheumatic drugs: Individually or in groups? A pilot randomized controlled trial comparing adherence and satisfaction. Musculoskeletal Care. 2009;7(2):78-92.
51. Hornnes N, Larsen K, Boysen G. Blood pressure 1 year after stroke: the need to optimize secondary prevention. J Stroke Cerebrovasc Dis. 2011;20(1):16-23.
52. Hunt JS, Siemienczuk J, Pape G, Rozenfeld Y, MacKay J, LeBlanc BH, et al. A randomized controlled trial of team-based care: impact of physician-pharmacist collaboration on uncontrolled hypertension. J Gen Intern Med. 2008;23(12):1966-72.
53. Johnson MO, Charlebois E, Morin SF, Remien RH, Chesney MA. Effects of a behavioral intervention on antiretroviral medication adherence among people living with HIV: the healthy living project randomized controlled study. J Acquir Immune Defic Syndr. 2007;46(5):574-80.
54. Johnson MO, Dilworth SE, Taylor JM, Neilands TB. Improving coping skills for self-management of treatment side effects can reduce antiretroviral medication nonadherence among people living with HIV. Ann Behav Med. 2011;41(1):83-91.
55. Joost R, Dorje F, Schwitulla J, Eckardt KU, Hugo C. Intensified pharmaceutical care is improving immunosuppressive medication adherence in kidney transplant recipients during the first post-transplant year: a quasi-experimental study. Nephrol Dial Transplant. 2014;29(8):1597-607.
56. Kellaway GS, McCrae E. The effect of counselling on compliance-failure in patient drug therapy. N Z Med J. 1979;89(631):161-5.
57. Kertes J, Dushenat M, Vesterman JL, Lemberger J, Bregman J, Friedman N. Factors contributing to compliance with osteoporosis medication. Isr Med Assoc J. 2008;10(3):207-13.
58. Kim KB, Han HR, Huh B, Nguyen T, Lee H, Kim MT. The effect of a community-based self-help multimodal behavioral intervention in Korean American seniors with high blood pressure. Am J Hypertens. 2014;27(9):1199-208.
59. Kim MT, Kim EY, Han HR, Jeong S, Lee JE, Park HJ, et al. Mail education is as effective as in-class education in hypertensive Korean patients. J Clin Hypertens (Greenwich). 2008;10(3):176-84.
60. Kiweewa FM, Wabwire D, Nakibuuka J, Mubiru M, Bagenda D, Musoke P, et al. Noninferiority of a task-shifting HIV care and treatment model using peer counselors and nurses among Ugandan women initiated on ART: evidence from a randomized trial. J Acquir Immune Defic Syndr. 2013;63(4):e125-32.
61. Klein A, Otto G, Kramer I. Impact of a pharmaceutical care program on liver transplant patients' compliance with immunosuppressive medication: a prospective, randomized, controlled trial using electronic monitoring. Transplantation. 2009;87(6):839-47.
62. Kooy MJ, van Wijk BLG, Heerdink ER, de Boer A, Bouvy ML. Does the use of an electronic reminder device with or without counseling improve adherence to lipid-lowering treatment? The results of a randomized controlled trial. Frontiers in Pharmacology. 2013;4:69.
63. Kripalani S, Schmotzer B, Jacobson TA. Improving Medication Adherence through Graphically Enhanced Interventions in Coronary Heart Disease (IMAGE-CHD): a randomized controlled trial. J Gen Intern Med. 2012;27(12):1609-17.
64. Lam DH, Watkins ER, Hayward P, Bright J, Wright K, Kerr N, et al. A randomized controlled study of cognitive therapy for relapse prevention for bipolar affective disorder: outcome of the first year. Arch Gen Psychiatry. 2003;60(2):145-52.
65. Lavoie KL, Moullec G, Lemiere C, Blais L, Labrecque M, Beauchesne MF, et al. Efficacy of brief motivational interviewing to improve adherence to inhaled corticosteroids among adult asthmatics: results from a randomized controlled pilot feasibility trial. Patient Prefer Adherence. 2014;8:1555-69.
66. Lee JK, Grace KA, Taylor AJ. Effect of a pharmacy care program on medication adherence and persistence, blood pressure, and low-density lipoprotein cholesterol: a randomized controlled trial. JAMA. 2006;296(21):2563-71.
67. Lester RT, Ritvo P, Mills EJ, Kariri A, Karanja S, Chung MH, et al. Effects of a mobile phone short message service on antiretroviral treatment adherence in Kenya (WelTel Kenya1): a randomised trial. Lancet. 2010;376(9755):1838-45.
68. Lin EH, Von Korff M, Ciechanowski P, Peterson D, Ludman EJ, Rutter CM, et al. Treatment adjustment and medication adherence for complex patients with diabetes, heart disease, and depression: a randomized controlled trial. Ann Fam Med. 2012;10(1):6-14.
69. Lopez Cabezas C, Falces Salvador C, Cubi Quadrada D, Arnau Bartes A, Ylla Bore M, Muro Perea N, et al. Randomized clinical trial of a postdischarge pharmaceutical care program vs regular follow-up in patients with heart failure. Farm Hosp. 2006;30(6):328-42.
70. Lucas GM, Mullen BA, Galai N, Moore RD, Cook K, McCaul ME, et al. Directly administered antiretroviral therapy for HIV-infected individuals in opioid treatment programs: results from a randomized clinical trial. PLoS One. 2013;8(7):e68286.
71. Ma Y, Ockene IS, Rosal MC, Merriam PA, Ockene JK, Gandhi PJ. Randomized Trial of a Pharmacist-Delivered Intervention for Improving Lipid-Lowering Medication Adherence among Patients with Coronary Heart Disease. Cholesterol. 2010;2010:383281.
72. Malotte CK, Hollingshead JR, Larro M. Incentives vs outreach workers for latent tuberculosis treatment in drug users. Am J Prev Med. 2001;20(2):103-7.
73. Migneault JP, Dedier JJ, Wright JA, Heeren T, Campbell MK, Morisky DE, et al. A culturally adapted telecommunication system to improve physical activity, diet quality, and medication adherence among hypertensive African-Americans: a randomized controlled trial. Ann Behav Med. 2012;43(1):62-73.
74. Molina JM, Podsadecki TJ, Johnson MA, Wilkin A, Domingo P, Myers R, et al. A lopinavir/ritonavir-based once-daily regimen results in better compliance and is non-inferior to a twice-daily regimen through 96 weeks. AIDS Res Hum Retroviruses. 2007;23(12):1505-14.
75. Morisky DE, DeMuth NM, Field-Fass M, Green LW, Levine DM. Evaluation of family health education to build social support for long-term control of high blood pressure. Health Educ Q. 1985;12(1):35-50.
76. Morisky DE, Malotte CK, Choi P, Davidson P, Rigler S, Sugland B, et al. A patient education program to improve adherence rates with antituberculosis drug regimens. Health Educ Q. 1990;17(3):253-67.
77. Moshkovska T, Stone MA, Smith RM, Bankart J, Baker R, Mayberry JF. Impact of a tailored patient preference intervention in adherence to 5-aminosalicylic acid medication in ulcerative colitis: results from an exploratory randomized controlled trial. Inflamm Bowel Dis. 2011;17(9):1874-81.
78. Mugusi F, Mugusi S, Bakari M, Hejdemann B, Josiah R, Janabi M, et al. Enhancing adherence to antiretroviral therapy at the HIV clinic in resource constrained countries; the Tanzanian experience. Trop Med Int Health. 2009;14(10):1226-32.
79. Munoz M, Finnegan K, Zeladita J, Caldas A, Sanchez E, Callacna M, et al. Community-based DOT-HAART accompaniment in an urban resource-poor setting. AIDS Behav. 2010;14(3):721-30.
80. Murray MD, Young J, Hoke S, Tu W, Weiner M, Morrow D, et al. Pharmacist intervention to improve medication adherence in heart failure: a randomized trial. Ann Intern Med. 2007;146(10):714-25.
81. Nesari M, Zakerimoghadam M, Rajab A, Bassampour S, Faghihzadeh S. Effect of telephone follow-up on adherence to a diabetes therapeutic regimen. Jpn J Nurs Sci. 2010;7(2):121-8.
82. Nielsen D, Ryg J, Nielsen W, Knold B, Nissen N, Brixen K. Patient education in groups increases knowledge of osteoporosis and adherence to treatment: a two-year randomized controlled trial. Patient Educ Couns. 2010;81(2):155-60.
83. Nieuwkerk PT, Nierman MC, Vissers MN, Locadia M, Greggers-Peusch P, Knape LP, et al. Intervention to improve adherence to lipid-lowering medication and lipid-levels in patients with an increased cardiovascular risk. Am J Cardiol. 2012;110(5):666-72.
84. O'Connor PJ, Schmittdiel JA, Pathak RD, Harris RI, Newton KM, Ohnsorg KA, et al. Randomized trial of telephone outreach to improve medication adherence and metabolic control in adults with diabetes. Diabetes Care. 2014;37(12):3317-24.
85. Odegard PS, Christensen DB. MAP study: RCT of a medication adherence program for patients with type 2 diabetes. J Am Pharm Assoc (2003). 2012;52(6):753-62.
86. Ogedegbe G, Chaplin W, Schoenthaler A, Statman D, Berger D, Richardson T, et al. A practice-based trial of motivational interviewing and adherence in hypertensive African Americans. Am J Hypertens. 2008;21(10):1137-43.
87. Ogedegbe GO, Boutin-Foster C, Wells MT, Allegrante JP, Isen AM, Jobe JB, et al. A randomized controlled trial of positive-affect intervention and medication adherence in hypertensive African Americans. Arch Intern Med. 2012;172(4):322-6.
88. Ogedegbe G, Tobin JN, Fernandez S, Cassells A, Diaz-Gloster M, Khalida C, et al. Counseling African Americans to Control Hypertension (CAATCH): Cluster Randomized Clinical Trial Main Effects. Circulation. 2014;129(20):2044-51.
89. Pagoto SL, McDermott MM, Reed G, Greenland P, Mazor KM, Ockene JK, et al. Can attention control conditions have detrimental effects on behavioral medicine randomized trials? Psychosom Med. 2013;75(2):137-43.
90. Palacio AM, Uribe C, Hazel-Fernandez L, Li H, Tamariz LJ, Garay SD, et al. Can phone-based motivational interviewing improve medication adherence to antiplatelet medications after a coronary stent among racial minorities? A randomized trial. J Gen Intern Med. 2015;30(4):469-75.
91. Pearson CR, Micek MA, Simoni JM, Hoff PD, Matediana E, Martin DP, et al. Randomized control trial of peer-delivered, modified directly observed therapy for HAART in Mozambique. J Acquir Immune Defic Syndr. 2007;46(2):238-44.
92. Piette JD, Weinberger M, Kraemer FB, McPhee SJ. Impact of automated calls with nurse follow-up on diabetes treatment outcomes in a Department of Veterans Affairs Health Care System: a randomized controlled trial. Diabetes Care. 2001;24(2):202-8.
93. Piette JD, Weinberger M, McPhee SJ, Mah CA, Kraemer FB, Crapo LM. Do automated calls with nurse follow-up improve self-care and glycemic control among vulnerable patients with diabetes? Am J Med. 2000;108(1):20-7.
94. Pladevall M, Divine G, Wells KE, Resnicow K, Williams LK. A randomized controlled trial to provide adherence information and motivational interviewing to improve diabetes and lipid control. Diabetes Educ. 2015;41(1):136-46.
95. Pop-Eleches C, Thirumurthy H, Habyarimana JP, Zivin JG, Goldstein MP, de Walque D, et al. Mobile phone technologies improve adherence to antiretroviral treatment in a resource-limited setting: a randomized controlled trial of text message reminders. AIDS. 2011;25(6):825-34.
96. Purcell DW, Latka MH, Metsch LR, Latkin CA, Gomez CA, Mizuno Y, et al. Results from a randomized controlled trial of a peer-mentoring intervention to reduce HIV transmission and increase access to care and adherence to HIV medications among HIV-seropositive injection drug users. J Acquir Immune Defic Syndr. 2007;46 Suppl 2:S35-47.
97. Pyne JM, Fortney JC, Curran GM, Tripathi S, Atkinson JH, Kilbourne AM, et al. Effectiveness of collaborative care for depression in human immunodeficiency virus clinics. Arch Intern Med. 2011;171(1):23-31.
98. Rabenda V, Mertens R, Fabri V, Vanoverloop J, Sumkay F, Vannecke C, et al. Adherence to bisphosphonates therapy and hip fracture risk in osteoporotic women. Osteoporos Int. 2008;19(6):811-8.
99. Rabenda V, Vanoverloop J, Fabri V, Mertens R, Sumkay F, Vannecke C, et al. Low incidence of anti-osteoporosis treatment after hip fracture. J Bone Joint Surg Am. 2008;90(10):2142-8.
100. Rea MM, Tompson MC, Miklowitz DJ, Goldstein MJ, Hwang S, Mintz J. Family-focused treatment versus individual treatment for bipolar disorder: results of a randomized clinical trial. J Consult Clin Psychol. 2003;71(3):482-92.
101. Reinares M, Colom F, Sanchez-Moreno J, Torrent C, Martinez-Aran A, Comes M, et al. Impact of caregiver group psychoeducation on the course and outcome of bipolar patients in remission: a randomized controlled trial. Bipolar Disord. 2008;10(4):511-9.
102. Reynolds NR, Testa MA, Su M, Chesney MA, Neidig JL, Frank I, et al. Telephone support to improve antiretroviral medication adherence: a multisite, randomized controlled trial. J Acquir Immune Defic Syndr. 2008;47(1):62-8.
103. Rinfret S, Lussier MT, Peirce A, Duhamel F, Cossette S, Lalonde L, et al. The impact of a multidisciplinary information technology-supported program on blood pressure control in primary care. Circ Cardiovasc Qual Outcomes. 2009;2(3):170-7.
104. Rinfret S, Rodes-Cabau J, Bagur R, Dery JP, Dorais M, Larose E, et al. Telephone contact to improve adherence to dual antiplatelet therapy after drug-eluting stent implantation. Heart. 2013;99(8):562-9.
105. Ringer J. Patient Persistency Project. JOURNALOFMANAGEDCAREPHARMAC~. 2001;7(1):50.
106. Robbins GK, Testa MA, Su M, Safren SA, Morse G, Lammert S, et al. Site nurse-initiated adherence and symptom support telephone calls for HIV-positive individuals starting antiretroviral therapy, ACTG 5031: substudy of ACTG 384. HIV Clin Trials. 2013;14(5):235-53.
107. Robinson JD, Segal R, Lopez LM, Doty RE. Impact of a pharmaceutical care intervention on blood pressure control in a chain pharmacy practice. Ann Pharmacother. 2010;44(1):88-96.
108. Sabin LL, DeSilva MB, Hamer DH, Xu K, Zhang J, Li T, et al. Using electronic drug monitor feedback to improve adherence to antiretroviral therapy among HIV-positive patients in China. AIDS Behav. 2010;14(3):580-9.
109. Sadik A, Yousif M, McElnay JC. Pharmaceutical care of patients with heart failure. Br J Clin Pharmacol. 2005;60(2):183-93.
110. Safren SA, O'Cleirigh C, Tan JY, Raminani SR, Reilly LC, Otto MW, et al. A randomized controlled trial of cognitive behavioral therapy for adherence and depression (CBT-AD) in HIV-infected individuals. Health Psychol. 2009;28(1):1-10.
111. Sajatovic M, Davies MA, Ganocy SJ, Bauer MS, Cassidy KA, Hays RW, et al. A comparison of the life goals program and treatment as usual for individuals with bipolar disorder. Psychiatr Serv. 2009;60(9):1182-9.
112. Samet JH, Horton NJ, Meli S, Dukes K, Tripps T, Sullivan L, et al. A randomized controlled trial to enhance antiretroviral therapy adherence in patients with a history of alcohol problems. Antivir Ther. 2005;10(1):83-93.
113. Sampaio-Sa M, Page-Shafer K, Bangsberg DR, Evans J, de Lourdes Dourado M, Teixeira C, et al. 100% adherence study: educational workshops vs. video sessions to improve adherence among ART-naive patients in Salvador, Brazil. AIDS and Behavior. 2008;12(1):54-62.
114. Sarna A, Luchters S, Geibel S, Chersich MF, Munyao P, Kaai S, et al. Short- and long-term efficacy of modified directly observed antiretroviral treatment in Mombasa, Kenya: a randomized trial. J Acquir Immune Defic Syndr. 2008;48(5):611-9.
115. Schneider PJ, Murphy JE, Pedersen CA. Impact of medication packaging on adherence and treatment outcomes in older ambulatory patients. J Am Pharm Assoc (2003). 2008;48(1):58-63.
116. Selke HM, Kimaiyo S, Sidle JE, Vedanthan R, Tierney WM, Shen C, et al. Task-shifting of antiretroviral delivery from health care workers to persons living with HIV/AIDS: clinical outcomes of a community-based program in Kenya. J Acquir Immune Defic Syndr. 2010;55(4):483-90.
117. Sewerynek E, Horst-Sikorska H, Stepien-Klos W, Antkowiak A, Janik M, Cieslak K, et al. The role of counselling and other factors in compliance of postmenopausal osteoporotic patients to alendronate 70 therapy. Arch Med Sci. 2013;9(2):288-96.
118. Shet A, De Costa A, Kumarasamy N, Rodrigues R, Rewari BB, Ashorn P, et al. Effect of mobile telephone reminders on treatment outcome in HIV: evidence from a randomised controlled trial in India. BMJ. 2014;349:g5978.
119. Silveira MP, Guttier MC, Page K, Moreira LB. Randomized controlled trial to evaluate the impact of pharmaceutical care on therapeutic success in HIV-infected patients in Southern Brazil. AIDS Behav. 2014;18 Suppl 1:S75-84.
120. Skaer TL, Sclar DA, Markowski DJ, Won JK. Effect of value-added utilities on prescription refill compliance and health care expenditures for hypertension. J Hum Hypertens. 1993;7(5):515-8.
121. Skaer TL, Sclar DA, Markowski DJ, Won JK. Effect of value-added utilities on prescription refill compliance and Medicaid health care expenditures--a study of patients with non-insulin-dependent diabetes mellitus. J Clin Pharm Ther. 1993;18(4):295-9.
122. Solomon DH, Iversen MD, Avorn J, Gleeson T, Brookhart MA, Patrick AR, et al. Osteoporosis Telephonic Intervention to Improve Medication Adherence (OPTIMA): A Large Pragmatic Randomized Controlled Trial. Archives of internal medicine. 2012;172(6):477-83.
123. Sosa N, Hill-Zabala C, Dejesus E, Herrera G, Florance A, Watson M, et al. Abacavir and lamivudine fixed-dose combination tablet once daily compared with abacavir and lamivudine twice daily in HIV-infected patients over 48 weeks (ESS30008, SEAL). J Acquir Immune Defic Syndr. 2005;40(4):422-7.
124. Staring AB, Van der Gaag M, Koopmans GT, Selten JP, Van Beveren JM, Hengeveld MW, et al. Treatment adherence therapy in people with psychotic disorders: randomised controlled trial. Br J Psychiatry. 2010;197(6):448-55.
125. Su WJ, Perng RP. Fixed-dose combination chemotherapy (Rifater/Rifinah) for active pulmonary tuberculosis in Taiwan: a two-year follow-up. Int J Tuberc Lung Dis. 2002;6(11):1029-32.
126. Taiwo BO, Idoko JA, Welty LJ, Otoh I, Job G, Iyaji PG, et al. Assessing the viorologic and adherence benefits of patient-selected HIV treatment partners in a resource-limited setting. J Acquir Immune Defic Syndr. 2010;54(1):85-92.
127. Tan H, Yu J, Tabby D, Devries A, Singer J. Clinical and economic impact of a specialty care management program among patients with multiple sclerosis: a cohort study. Mult Scler. 2010;16(8):956-63.
128. Taylor CT, Byrd DC, Krueger K. Improving primary care in rural Alabama with a pharmacy initiative. Am J Health Syst Pharm. 2003;60(11):1123-9.
129. Thom S, Poulter N, Field J, Patel A, Prabhakaran D, Stanton A, et al. Effects of a fixed-dose combination strategy on adherence and risk factors in patients with or at high risk of CVD: the UMPIRE randomized clinical trial. JAMA. 2013;310(9):918-29.
130. Tinsel I, Buchholz A, Vach W, Siegel A, Durk T, Buchholz A, et al. Shared decision-making in antihypertensive therapy: a cluster randomised controlled trial. BMC Fam Pract. 2013;14:135.
131. Tuldra A, Fumaz CR, Ferrer MJ, Bayes R, Arno A, Balague M, et al. Prospective randomized two-Arm controlled study to determine the efficacy of a specific intervention to improve long-term adherence to highly active antiretroviral therapy. J Acquir Immune Defic Syndr. 2000;25(3):221-8.
132. Valencia M, Rascon ML, Juarez F, Murow E. A psychosocial skills training approach in Mexican out-patients with schizophrenia. Psychol Med. 2007;37(10):1393-402.
133. van Onzenoort HA, Verberk WJ, Kroon AA, Kessels AG, Nelemans PJ, van der Kuy PH, et al. Effect of self-measurement of blood pressure on adherence to treatment in patients with mild-to-moderate hypertension. J Hypertens. 2010;28(3):622-7.
134. Varma S, McElnay JC, Hughes CM, Passmore AP, Varma M. Pharmaceutical care of patients with congestive heart failure: interventions and outcomes. Pharmacotherapy. 1999;19(7):860-9.
135. Velligan DI, Diamond PM, Mintz J, Maples N, Li X, Zeber J, et al. The use of individually tailored environmental supports to improve medication adherence and outcomes in schizophrenia. Schizophr Bull. 2008;34(3):483-93.
136. Villeneuve J, Genest J, Blais L, Vanier MC, Lamarre D, Fredette M, et al. A cluster randomized controlled Trial to Evaluate an Ambulatory primary care Management program for patients with dyslipidemia: the TEAM study. CMAJ. 2010;182(5):447-55.
137. Vollmer WM, Owen-Smith AA, Tom JO, Laws R, Ditmer DG, Smith DH, et al. Improving adherence to cardiovascular disease medications with information technology. Am J Manag Care. 2014;20(11 Spec No. 17):SP502-10.
138. Vrijens B, Belmans A, Matthys K, de Klerk E, Lesaffre E. Effect of intervention through a pharmaceutical care program on patient adherence with prescribed once-daily atorvastatin. Pharmacoepidemiol Drug Saf. 2006;15(2):115-21.
139. Wagner GJ, Kanouse DE, Golinelli D, Miller LG, Daar ES, Witt MD, et al. Cognitive-behavioral intervention to enhance adherence to antiretroviral therapy: a randomized controlled trial (CCTG 578). AIDS. 2006;20(9):1295-302.
140. Wakefield BJ, Holman JE, Ray A, Scherubel M, Adams MR, Hills SL, et al. Outcomes of a home telehealth intervention for patients with diabetes and hypertension. Telemed J E Health. 2012;18(8):575-9.
141. Wang J, Wu J, Yang J, Zhuang Y, Chen J, Qian W, et al. Effects of pharmaceutical care interventions on blood pressure and medication adherence of patients with primary hypertension in China. Clinical Research and Regulatory Affairs. 2011;28(1):1-6.
142. Webb PA. Effectiveness of patient education and psychosocial counseling in promoting compliance and control among hypertensive patients. J Fam Pract. 1980;10(6):1047-55.
143. Weber R, Christen L, Christen S, Tschopp S, Znoj H, Schneider C, et al. Effect of individual cognitive behaviour intervention on adherence to antiretroviral therapy: prospective randomized trial. Antivir Ther. 2004;9(1):85-95.
144. Weinberger M, Tierney WM, Booher P, Katz BP. The impact of increased contact on psychosocial outcomes in patients with osteoarthritis: a randomized, controlled trial. J Rheumatol. 1991;18(6):849-54.
145. Williams AB, Fennie KP, Bova CA, Burgess JD, Danvers KA, Dieckhaus KD. Home visits to improve adherence to highly active antiretroviral therapy: a randomized controlled trial. J Acquir Immune Defic Syndr. 2006;42(3):314-21.
146. Williams AB, Wang H, Li X, Chen J, Li L, Fennie K. Efficacy of an evidence-based ARV adherence intervention in China. AIDS Patient Care STDS. 2014;28(8):411-7.
147. Windsor RA, Bailey WC, Richards JM, Jr., Manzella B, Soong SJ, Brooks M. Evaluation of the efficacy and cost effectiveness of health education methods to increase medication adherence among adults with asthma. Am J Public Health. 1990;80(12):1519-21.
148. Zillich AJ, Jaynes HA, Snyder ME, Harrison J, Hudmon KS, de Moor C, et al. Evaluation of specialized medication packaging combined with medication therapy management: adherence, outcomes, and costs among Medicaid patients. Med Care. 2012;50(6):485-93.
149. Zwikker HE, van den Ende CH, van Lankveld WG, den Broeder AA, van den Hoogen FH, van de Mosselaar B, et al. Effectiveness of a group-based intervention to change medication beliefs and improve medication adherence in patients with rheumatoid arthritis: a randomized controlled trial. Patient Educ Couns. 2014;94(3):356-61.
     1. **Excluded primary studies**

| **Meta-analysis** | **Original studies** |
| --- | --- |
| Easthall C, Song F, Bhattacharya D. A meta-analysis of cognitive-based behaviour change techniques as interventions to improve medication adherence. BMJ Open. 2013;3(8). | |
|  | Berger, 2005 |
|  | Brown, 2009 |
|  | George, 2010 |
|  | Hovell, 2003 |
|  | Maneesriwongul, 2012 |
|  | Put, 2003 |
|  | Sheeran, 1999 |
|  | Smith, 2003 |
|  | Solomon, 2012 |
|  | Van Es, 2001 |
|  | Williams, 2012 |
| Tao D, Xie L, Wang T, Wang T. A meta-analysis of the use of electronic reminders for patient adherence to medication in chronic disease care. J Telemed Telecare. 2015;21(1):3-13. | |
|  | Andrade, 2005 |
|  | Charles, 2007 |
|  | Christensen, 2010 |
|  | Chung, 2011 |
|  | Franklin, 2006 |
|  | Ho, 2008 |
|  | Petrie, 2012 |
|  | Simoni, 2009 |
| Sakakibara BM, Kim AJ, Eng JJ. A Systematic Review and Meta-Analysis on Self-Management for Improving Risk Factor Control in Stroke Patients. Int J Behav Med. 2017;24(1):42-53. | |
|  | Evans-Hudnall, 2014 |
|  | Kim, 2013 |
|  | Kronish, 2014 |
|  | Mackenzie, 2013 |
|  | O'Carroll, 2013 |
| Rash JA, Campbell DJ, Tonelli M, Campbell TS. A systematic review of interventions to improve adherence to statin medication: What do we know about what works? Prev Med. 2016;90:155-69. | |
|  | Aslani, 2011 |
|  | Evans, 2010 |
|  | Flemming, 2013 |
|  | Jarab, 2012 |
|  | Kardas, 2012 |
|  | Mols, 2015 |
|  | Patel, 2015 |
|  | van Bruggen, 2009 |
| Kassavou A, Sutton S. Automated telecommunication interventions to promote adherence to cardio-metabolic medications: meta-analysis of effectiveness and meta-regression of behaviour change techniques. Health Psychol Rev. 2018;12(1):25-42. | |
|  | Arora, 2014 |
|  | Gatwood, 2016 |
|  | Kamal, 2015 |
|  | Sherrard, 2015 |
|  | Stacy, 2009 |
| Parienti JJ, Bangsberg DR, Verdon R, Gardner EM. Better adherence with once-daily antiretroviral regimens: a meta-analysis. Clin Infect Dis. 2009;48(4):484-8. | |
|  | Benson, 2004 |
|  | Kubota, 2006 |
|  | Parienti, 2007 |
|  | Portsmouth, 2005 |
|  | Rode, 2008 |
|  | Ruane, 2006 |
| Falagas ME, Karagiannis AK, Nakouti T, Tansarli GS. Compliance with once-daily versus twice or thrice-daily administration of antibiotic regimens: a meta-analysis of randomized controlled trials. PLoS One. 2015;10(1):e0116207. | |
|  | Adam, 2001 |
|  | Ballantyne, 1985 |
|  | Behre, 1997 |
|  | Cheung, 1988 |
|  | Clegg, 2006 |
|  | Cohen, 1996 |
|  | Cook, 1996 |
|  | Damrikarnlert, 2000 |
|  | Disney, 1990 |
|  | Edelstein, 1993 |
|  | Fyllingen, 1991 |
|  | Garcia Callejo, 1998 |
|  | Gehanno, 1994 |
|  | Gerber, 1985 |
|  | Gooch, 1993 |
|  | Gooch, 1997 |
|  | Hosie, 1995 |
|  | Kardas, 2007 |
|  | Lennon, 2008 |
|  | Linder, 1993 |
|  | Martinot, 2001 |
|  | Mita, 2003 |
|  | Owen, 1993 |
|  | Pichichero, 1999 |
|  | Richard, 1981 |
|  | Venuta, 1998 |
| Conn VS, Enriquez M, Ruppar TM, Chan KC. Cultural relevance in medication adherence interventions with underrepresented adults: systematic review and meta-analysis of outcomes. Prev Med. 2014;69:239-47. | |
|  | Anderson, 2004 |
|  | Austin, 1986 |
|  | Babamoto, 2009 |
|  | Bogart, 2012 |
|  | Bogner, 2008 |
|  | Chaisson, 2001 |
|  | Cordasco, 2009 |
|  | Fernandez, 2008 |
|  | Fisher, 2011 |
|  | Freedman, 2007 |
|  | Gazmararian, 2010 |
|  | Harper, 1984 |
|  | Laine, 1996 |
|  | Mann, 2001 |
|  | Martin, 2011 |
|  | McKenney, 1973 |
|  | McKenney, 1978 |
|  | Moitra, 2011 |
|  | Muir, 2012 |
|  | Mundy, 2009 |
|  | Newsome, 1995 |
|  | Powell, 2002 |
|  | Tagliacozzo, 1974 |
|  | Vivian, 2002 |
|  | Walker, 2000 |
|  | Webel, 2010 |
|  | Werner, 1979 |
|  | Wyatt, 2004 |
| Hart JE, Jeon CY, Ivers LC, Behforouz HL, Caldas A, Drobac PC, et al. Effect of directly observed therapy for highly active antiretroviral therapy on virologic, immunologic, and adherence outcomes: a meta-analysis and systematic review. J Acquir Immune Defic Syndr. 2010;54(2):167-79. | |
|  | Bangsberg, 2009 |
| Mutasa-Apollo T, Ford N, Wiens M, Socias ME, Negussie E, Wu P, et al. Effect of frequency of clinic visits and medication pick-up on antiretroviral treatment outcomes: a systematic literature review and meta-analysis. J Int AIDS Soc. 2017;20(Suppl 4):21647. | |
|  | Babigumira, 2011 |
|  | Jaffar, 2009 |
| Readdean KC, Heuer AJ, Scott Parrott J. Effect of pharmacist intervention on improving antidepressant medication adherence and depression symptomology: A systematic review and meta-analysis. Res Social Adm Pharm. 2017. | |
|  | Aljumah, 2015 |
|  | Al-Saffar, 2005 |
|  | Finley, 2002 |
|  | Klang, 2015 |
|  | Rubio-Valera, 2013 |
| Morrissey EC, Durand H, Nieuwlaat R, Navarro T, Haynes RB, Walsh JC, et al. Effectiveness and content analysis of interventions to enhance medication adherence and blood pressure control in hypertension: A systematic review and meta-analysis. Psychol Health. 2017;32(10):1195-232. | |
|  | Dusing, 2009 |
|  | Girvin, 1999 |
|  | Greer, 2014 |
|  | Margolius, 2012 |
|  | Matsumura, 2012 |
|  | Sackett, 1975 |
|  | Stewart, 2014 |
| Zomahoun HTV, Guenette L, Gregoire JP, Lauzier S, Lawani AM, Ferdynus C, et al. Effectiveness of motivational interviewing interventions on medication adherence in adults with chronic diseases: a systematic review and meta-analysis. Int J Epidemiol. | |
|  | DiIorio, 2009 |
|  | Kender, 2010 |
|  | Martino, 2006 |
|  | Williams-a, 2012 |
| Rubio-Valera M, Serrano-Blanco A, Magdalena-Belio J, Fernandez A, Garcia-Campayo J, Pujol MM, et al. Effectiveness of pharmacist care in the improvement of adherence to antidepressants: a systematic review and meta-analysis. Ann Pharmacother. 2011;45(1):39-48. | |
|  | Adler, 2004 |
|  | Brook, 2005 |
|  | Crockett, 2006 |
|  | Finley, 2003 |
|  | Rickles, 2005 |
| Vignon Zomahoun HT, de Bruin M, Guillaumie L, Moisan J, Gregoire JP, Perez N, et al. Effectiveness and Content Analysis of Interventions to Enhance Oral Antidiabetic Drug Adherence in Adults with Type 2 Diabetes: Systematic Review and Meta-Analysis. Value Health. 2015;18(4):530-40. | |
|  | Bogner, 2010 |
|  | Bogner, 2012 |
|  | Farmer, 2012 |
|  | Heisler, 2010 |
|  | Rothschild, 2014 |
|  | Walker, 2011 |
|  | Zolfaghari, 2012 |
| Zhu Y, Zhou Y, Zhang L, Zhang J, Lin J. Efficacy of interventions for adherence to the immunosuppressive therapy in kidney transplant recipients: a meta-analysis and systematic review. J Investig Med. 2017;65(7):1049-56. | |
|  | Bessa, 2016 |
|  | Chisholm-Burns, 2013 |
|  | Fennell, 1994 |
|  | Garcia, 2015 |
| Simoni JM, Pearson CR, Pantalone DW, Marks G, Crepaz N. Efficacy of interventions in improving highly active antiretroviral therapy adherence and HIV-1 RNA viral load. A meta-analytic review of randomized controlled trials. J Acquir Immune Defic Syndr. 2006;43 Suppl 1:S23-35. | |
|  | Jones, 2003 |
|  | Knobel, 1999 |
|  | Margolin, 2003 |
|  | Rawlings, 2003 |
|  | Rotheram-Borus, 2004 |
| Langebeek N, Nieuwkerk P. Electronic medication monitoring-informed counseling to improve adherence to combination anti-retroviral therapy and virologic treatment outcomes: a meta-analysis. Front Public Health. 2015;3:139. | |
|  | Davies, 2010 |
|  | Engelbrecht, 2010 |
|  | Sabin, 2014 |
|  | Wilson, 2010 |
| Petry NM, Rash CJ, Byrne S, Ashraf S, White WB. Financial reinforcers for improving medication adherence: findings from a meta-analysis. Am J Med. 2012;125(9):888-96. | |
|  | Bock, 2001 |
|  | Carroll, 2001 |
|  | Carroll, 2002 |
|  | Claassen, 2007 |
|  | Grabowski, 1979 |
|  | Jakubowiak, 2007 |
|  | Martins, 2009 |
|  | Morisky, 2001 |
|  | Nunes, 2006 |
|  | Preston, 1999 |
|  | Rigsby, 2000 |
|  | Seal, 2003 |
|  | Stitzer, 2010 |
|  | Tulsky, 2000 |
|  | Volpp, 2008 |
| Bangalore S, Kamalakkannan G, Parkar S, Messerli FH. Fixed-dose combinations improve medication compliance: a meta-analysis. Am J Med. 2007;120(8):713-9. | |
|  | Dezii, 2000 |
|  | Melikian, 2002 |
|  | Melikian, 2002 |
|  | NDC data set |
| Demonceau J, Ruppar T, Kristanto P, Hughes DA, Fargher E, Kardas P, et al. Identification and assessment of adherence-enhancing interventions in studies assessing medication adherence through electronically compiled drug dosing histories: a systematic literature review and meta-analysis. Drugs. 2013;73(6):545-62. | |
|  | Apter, 2011 |
|  | Berg, 1997 |
|  | Berkovitch, 1998 |
|  | Cramer, 1999 |
|  | Ducharme, 2011 |
|  | Grosset, 2007 |
|  | Janson, 2003 |
|  | Kardas, 2004 |
|  | Kozuki, 2006 |
|  | Onyirimba, 2003 |
|  | Qureshi, 2007 |
|  | Rapoff, 2002 |
| Burudpakdee C, Khan ZM, Gala S, Nanavaty M, Kaura S. Impact of patient programs on adherence and persistence in inflammatory and immunologic diseases: a meta-analysis. Patient Prefer Adherence. 2015;9:435-48. | |
|  | Cook, 2010 |
|  | Elkjaer, 2010 |
|  | Heilmann, 2012 |
|  | Lai, 2010 |
|  | Montori, 2011 |
|  | Moss, 2010 |
|  | Shu, 2009 |
|  | Stockl, 2010 |
|  | Stockl, 2010b |
|  | Tamone, 2012 |
|  | Ting, 2012 |
| MacDonald L, Chapman S, Syrett M, Bowskill R, Horne R. Improving medication adherence in bipolar disorder: A systematic review and meta-analysis of 30 years of intervention trials. J Affect Disord. 2016;194:202-21. | |
|  | Castle, 2007 |
|  | Clarkin, 1998 |
|  | Cochran, 1984 |
|  | Colom, 2003 |
|  | Dogan, 2003 |
|  | Eker, 2012 |
|  | Elixhauser, 1990 |
|  | Lam, 2000 |
|  | Lenz, 2010 |
|  | Miklowitz, 2000 |
|  | van Gent, 1991 |
|  | Zaretsky, 2008 |
| Kauppi K, Valimaki M, Hatonen HM, Kuosmanen LM, Warwick-Smith K, Adams CE. Information and communication technology based prompting for treatment compliance for people with serious mental illness. Cochrane Database Syst Rev. 2014(6):CD009960. | |
|  | Hulsbosch, 2008 |
|  | Montes, 2011 |
| Kanters S, Park JJ, Chan K, Socias ME, Ford N, Forrest JI, et al. Interventions to improve adherence to antiretroviral therapy: a systematic review and network meta-analysis. Lancet HIV. 2017;4(1):e31-e40. | |
|  | Belzer, 2014 |
|  | Duncan, 2012 |
|  | Garcia, 2005 |
|  | Hersch, 2013 |
|  | Kurth, 2014 |
|  | Levin, 2006 |
|  | Naar-King, 2013 |
|  | Orrell, 2015 |
|  | Peltzer, 2012 |
|  | Safren, 2012 |
|  | Wagner, 2013 |
|  | Wamalwa, 2009 |
| Normansell R, Kew KM, Stovold E. Interventions to improve adherence to inhaled steroids for asthma. Cochrane Database Syst Rev. 2017;4:CD012226. | |
|  | Bender, 2014 |
|  | Chatkin, 2006 |
|  | Duncan, 2013 |
|  | Foster, 2014 |
|  | Hederos, 2005 |
|  | Mehuys, 2008 |
|  | Otsuki, 2009 |
| van Driel ML, Morledge MD, Ulep R, Shaffer JP, Davies P, Deichmann R. Interventions to improve adherence to lipid-lowering medication. Cochrane Database Syst Rev. 2016;12:CD004371. | |
|  | Marquez-Contreras, 2004 |
|  | Marquez-Contreras, 2007 |
| Conn VS, Hafdahl AR, Cooper PS, Ruppar TM, Mehr DR, Russell CL. Interventions to improve medication adherence among older adults: meta-analysis of adherence outcomes among randomized controlled trials. Gerontologist. 2009;49(4):447-62. | |
|  | Blenkinsopp, 2000 |
|  | Brun, 1994 |
|  | Edworthy, 1999 |
|  | Gabriel, 1977 |
|  | Grant, 2003 |
|  | Halfmann, 2000 |
|  | Jennings, 1992 |
|  | Klein, 2006 |
|  | Lipton, 1994 |
|  | Lourens, 1994 |
|  | Pullar, 1988 |
|  | Raynor, 1993 |
|  | Rimer, 1987 |
|  | Roden, 1985 |
|  | Rozenfeld, 1999 |
|  | Schectman, 1994 |
|  | Simkins, 1986 |
|  | Solomon, 1998 |
|  | Wood, 1989 |
| Santo K, Kirkendall S, Laba TL, Thakkar J, Webster R, Chalmers J, et al. Interventions to improve medication adherence in coronary disease patients: A systematic review and meta-analysis of randomised controlled trials. Eur J Prev Cardiol. 2016;23(10):1065-76. | |
|  | Calvert, 2012 |
|  | Castellano, 2014 |
|  | Gould, 2011 |
|  | Gould, 2011 |
|  | Jiang, 2007 |
|  | Lourenco, 2014 |
|  | Ostbring, 2014 |
|  | Polack, 2008 |
| Conn VS, Ruppar TM, Chase JA, Enriquez M, Cooper PS. Interventions to Improve Medication Adherence in Hypertensive Patients: Systematic Review and Meta-analysis. Curr Hypertens Rep. 2015;17(12):94. | |
|  | Adeyemo, 2013 |
|  | Aguwa, 2008 |
|  | Al Owaish, 1985 |
|  | Avanzini, 2002 |
|  | Beeson, 1977 |
|  | Beune, 2014 |
|  | Carter, 2010 |
|  | Cooper, 2011 |
|  | Erickson, 2003 |
|  | Evans, 1986 |
|  | Fletcher, 1975 |
|  | Friedberg, 2015 |
|  | Gomez-Marcos, 2006 |
|  | Gonzalez-Fernandez, 1990 |
|  | Hacihasanoglu, 2011 |
|  | Harowski, 1983 |
|  | Harper, 1984 |
|  | Hess, 2007 |
|  | Inui, 1976 |
|  | Jafar, 2009 |
|  | Kauric-Klein, 2012 |
|  | Kobalava, 2010 |
|  | Leung, 2012 |
|  | Levine, 1979 |
|  | Logan, 1982 |
|  | Ma, 2014 |
|  | Magadza, 2009 |
|  | Marquez-Contreras, 2005 |
|  | Martin, 2011 |
|  | McGillicuddy, 2013 |
|  | McKinstry, 2013 |
|  | Moore, 2013 |
|  | Morgado, 2011 |
|  | Muhlhauser, 1988 |
|  | Murray, 2004 |
|  | Oliver, 2011 |
|  | Oparah, 2006 |
|  | Park, 1996 |
|  | Park, 2013 |
|  | Patel, 2013 |
|  | Pertusa Martinez, 1998 |
|  | Pierce, 1984 |
|  | Pippalla, 1994 |
|  | Pladevall, 2010 |
|  | Planas, 2003 |
|  | Resnick, 2009 |
|  | Roca-Cusachs, 1991 |
|  | Rodriguez, 2011 |
|  | Roumie, 2006 |
|  | Saleem, 2013 |
|  | Santschi, 2008 |
|  | Schroeder, 2005 |
|  | Sicras-Mainar, 2013 |
|  | Sookaneknun, 2004 |
|  | Stewart, 2005 |
|  | Stewart, 2008 |
|  | Svarstad, 2013 |
|  | van de Steeg-van Gompel, 2010 |
|  | Wentzlaff, 2011 |
|  | Wong, 2013 |
|  | Zang, 2010 |
| Hollands GJ, McDermott MS, Lindson-Hawley N, Vogt F, Farley A, Aveyard P. Interventions to increase adherence to medications for tobacco dependence. Cochrane Database Syst Rev. 2015(2):CD009164. | |
|  | Marteau, 2012 |
|  | Mooney, 2005 |
|  | Mooney, 2007 |
|  | Nollen, 2011 |
|  | Schmitz, 2005 |
|  | Smith, 2013 |
| Gray R, Bressington D, Ivanecka A, Hardy S, Jones M, Schulz M, et al. Is adherence therapy an effective adjunct treatment for patients with schizophrenia spectrum disorders? A systematic review and meta-analysis. BMC Psychiatry. 2016;16:90. | |
|  | Anderson, 2010 |
|  | Chien, 2015 |
|  | Maneesakorn, 2007 |
|  | Schulz, 2013 |
|  | von Bormann, 2015 |
| Manias E, Williams A. Medication adherence in people of culturally and linguistically diverse backgrounds: a meta-analysis. Ann Pharmacother. 2010;44(6):964-82. | |
|  | Armour, 2004 |
|  | Berrien, 2004 |
|  | Bonner, 2002 |
|  | Canino, 2008 |
|  | Krier, 1999 |
|  | Morisky, 2001 |
|  | Rich, 1995 |
|  | Schaffer and Tian, 2004 |
|  | Schwartz-Lookinland, 1989 |
|  | van Servellen, 2003 |
|  | Yin, 2008 |
| Ruppar TM, Dunbar-Jacob JM, Mehr DR, Lewis L, Conn VS. Medication adherence interventions among hypertensive black adults: a systematic review and meta-analysis. J Hypertens. 2017;35(6):1145-54. | |
|  | Crowley, 2013 |
|  | Greer, 2011 |
| Ruppar TM, Delgado JM, Temple J. Medication adherence interventions for heart failure patients: A meta-analysis. Eur J Cardiovasc Nurs. 2015;14(5):395-404. | |
|  | Bouvy, 2003 |
|  | Dawson, 1998 |
|  | Goodyer, 1995 |
|  | Jerant, 2003 |
|  | Nimpitakpong, 2002 |
|  | Nucifora, 2006 |
|  | Powell, 2010 |
|  | Rich, 1996 |
|  | Tierney, 2003 |
|  | Udelson, 2009 |
|  | Wakefield, 2009 |
|  | Wu, 2012 |
| Conn VS, Ruppar TM, Enriquez M, Cooper P. Medication adherence interventions that target subjects with adherence problems: Systematic review and meta-analysis. Res Social Adm Pharm. 2016;12(2):218-46. | |
|  | Alhalaiqa, 2012 |
|  | Austin, 1986 |
|  | Cook, 2010 |
|  | De Geest, 2006 |
|  | Freedman, 2007 |
|  | Harper, 1984 |
|  | Haynes, 1976 |
|  | Kogos, 2004 |
|  | Matterson, 2011 |
|  | Mitchell, 1992 |
|  | Moitra, 2011 |
|  | Murphy, 2002 |
|  | Murphy, 2007 |
|  | Nietert, 2009 |
|  | Okeke, 2009 |
|  | Oser, 2008 |
|  | Ramirez-Garcia & Cote, 2012 |
|  | Remien, 2005 |
|  | Rosen, 2007 |
|  | Ruppar, 2010 |
|  | Russell, 2010 |
|  | Safren, 2003 |
|  | Sorensen, 2007 |
|  | Stewart, 2008 |
|  | Van Servellen, 2005 |
|  | Wall, 1995 |
|  | Watakakasol, 2010 |
|  | Wu, 2006 |
|  | Zuckerman, 2004 |
| Takiya LN, Peterson AM, Finley RS. Meta-analysis of interventions for medication adherence to antihypertensives. Ann Pharmacother. 2004;38(10):1617-24. | |
|  | Eisen, 1990 |
|  | Friedman, 1996 |
|  | Girvin, 1999 |
|  | Leenen, 1997 |
|  | Mehos, 2000 |
|  | Nessman, 1980 |
|  | Rehder, 1980 |
|  | Saunders, 1991 |
|  | Sclar, 1991 |
| Peterson AM, Takiya L, Finley R. Meta-analysis of trials of interventions to improve medication adherence. Am J Health Syst Pharm. 2003;60(7):657-65. | |
|  | Bertakis, 1986 |
|  | Brown, 1997 |
|  | Colcher, 1972 |
|  | Cole, 1971 |
|  | Cramer, 1995 |
|  | Ellison, 1982 |
|  | Eriksson, 1998 |
|  | Finney, 1985 |
|  | Garnett, 1981 |
|  | Gibbs, 1989 |
|  | Jameson, 1995 |
|  | Katon, 1996 |
|  | Kelly, 1990 |
|  | Kruse, 1991 |
|  | Levesque, 1983 |
|  | Linszen, 1996 |
|  | McCrindle, 1997 |
|  | Mengze, 1994 |
|  | Murray, 1993 |
|  | Powell, 1995 |
|  | Sanmarti, 1993 |
|  | Sellors, 1997 |
|  | Sharpe, 1974 |
|  | Smith, 1986 |
|  | Spriet, 1980 |
|  | Taggart, 1981 |
|  | Tinkelman, 1980 |
|  | Williams, 1986 |
| Miller L, Schuz B, Walters J, Walters EH. Mobile Technology Interventions for Asthma Self-Management: Systematic Review and Meta-Analysis. JMIR Mhealth Uhealth. 2017;5(5):e57. | |
|  | Liu, 2007 |
|  | Ostojic, 2005 |
|  | Ryan, 2012 |
| Thakkar J, Kurup R, Laba TL, Santo K, Thiagalingam A, Rodgers A, et al. Mobile Telephone Text Messaging for Medication Adherence in Chronic Disease: A Meta-analysis. JAMA Intern Med. 2016;176(3):340-9. | |
|  | da Costa, 2012 |
|  | Hardy, 2011 |
|  | Khonsari, 2015 |
|  | Lua and Neni, 2013 |
|  | Lv, 2012 |
|  | Maduka and Tobin-West, 2013 |
|  | Marquez Contreras, 2004 |
|  | Mbuagbaw, 2012 |
|  | Park, 2014 |
|  | Quilici, 2013 |
|  | Quilici, 2013 |
|  | Strandbygaard, 2010 |
|  | Vervloet, 2012 |
|  | Wald, 2014 |
|  | Wang, 2014 |
| Palacio A, Garay D, Langer B, Taylor J, Wood BA, Tamariz L. Motivational Interviewing Improves Medication Adherence: a Systematic Review and Meta-analysis. J Gen Intern Med. 2016;31(8):929-40. | |
|  | Berger, 2003 |
|  | DiIorio, 2008 |
|  | Golin, 2006 |
|  | Holstad, 2011 |
|  | Holstad, 2012 |
|  | Ingersoll, 2011 |
|  | Interian, 2013 |
|  | Konkle-Parker, 2012 |
|  | Parsons, 2007 |
|  | Pradier, 2003 |
| Van Camp YP, Van Rompaey B, Elseviers MM. Nurse-led interventions to enhance adherence to chronic medication: systematic review and meta-analysis of randomised controlled trials. Eur J Clin Pharmacol. 2013;69(4):761-70. | |
|  | de Bruin, 2010 |
|  | Holzemer, 2006 |
|  | Koenig, 2008 |
|  | Simon, 2011 |
|  | Simoni, 2011 |
| Wald DS, Butt S, Bestwick JP. One-way versus two-way text messaging on improving medication adherence: meta-analysis of randomized trials. Am J Med. 2015;128(10):1139 e1-5. | |
|  | Ollivier, 2009 |
| Conn VS, Ruppar TM, Chan KC, Dunbar-Jacob J, Pepper GA, De Geest S. Packaging interventions to increase medication adherence: systematic review and meta-analysis. Curr Med Res Opin. 2015;31(1):145-60. | |
|  | Awofeso, 1995 |
|  | Becker, 1986 |
|  | Bosworth, 2008 |
|  | Burelle, 1986 |
|  | Crome, 1982 |
|  | Eshelman, 1976 |
|  | Henry, 1999 |
|  | Kalichman, 2011 |
|  | Kennedy, 1990 |
|  | Laramee, 2003 |
|  | Lee, 1999 |
|  | Levensky, 2006 |
|  | Linkewich, 1974 |
|  | MacDonald, 1977 |
|  | MacIntosh, 2007 |
|  | McPherson-Baker, 2000 |
|  | Nazareth, 2001 |
|  | Park, 1992 |
|  | Peterson, 1984 |
|  | Qingjun, 1998a |
|  | Revankar, 1993 |
|  | Safren, 2001 |
|  | Spriet, 1980 |
|  | Suarez-Varela, 2009 |
|  | Sweeney, 1989 |
|  | Traiger, 1997 |
|  | Tsuyuki, 2004 |
|  | Wang, 2010 |
|  | Wright, 1999 |
|  | Zillich, 2005 |
| Rocha BS, Silveira MP, Moraes CG, Kuchenbecker RS, Dal-Pizzol TS. Pharmaceutical interventions in antiretroviral therapy: systematic review and meta-analysis of randomized clinical trials. J Clin Pharm Ther. 2015;40(3):251-8. | |
|  | Rathbun, 2005 |
| Iskedjian M, Einarson TR, MacKeigan LD, Shear N, Addis A, Mittmann N, et al. Relationship between daily dose frequency and adherence to antihypertensive pharmacotherapy: evidence from a meta-analysis. Clin Ther. 2002;24(2):302-16. | |
|  | Baird, 1984 |
|  | Boissel, 1996 |
|  | Detry, 1995 |
|  | Eisen, 1990 |
|  | Fujii, 1985 |
|  | Halpern, 1993 |
|  | Hilleman, 1993 |
| Heneghan CJ, Glasziou P, Perera R. Reminder packaging for improving adherence to self-administered long-term medications. Cochrane Database Syst Rev. 2006(1):CD005025. | |
| Mahtani KR, Heneghan CJ, Glasziou PP, Perera R. Reminder packaging for improving adherence to self-administered long-term medications. Cochrane Database Syst Rev. 2011(9):CD005025. | |
|  | Azrin, 1998 |
|  | Binstock, 1988 |
|  | Huang, 2000 |
|  | Jansen, 2009 |
|  | Kripalani, 2007 |
|  | Simmons, 2000 |
|  | Suppapitiporn, 2005 |
|  | Winland-Brown, 2000 |
| Zou H, Li Z, Nolan MT, Arthur D, Wang H, Hu L. Self-management education interventions for persons with schizophrenia: a meta-analysis. Int J Ment Health Nurs. 2013;22(3):256-71. | |
|  | Hornung, 1996 |
|  | Kopelowicz, 2003 |
|  | Pitschel-Walz, 2006 |
| de Bruin M, Viechtbauer W, Schaalma HP, Kok G, Abraham C, Hospers HJ. Standard care impact on effects of highly active antiretroviral therapy adherence interventions: A meta-analysis of randomized controlled trials. Arch Intern Med. 2010;170(3):240-50. | |
|  | Jones, 2007 |
|  | Simoni, 2007 |
|  | Tuldra, 2002 |
|  | Wohl, 2006 |
| Iglay K, Cao X, Mavros P, Joshi K, Yu S, Tunceli K. Systematic Literature Review and Meta-analysis of Medication Adherence With Once-weekly Versus Once-daily Therapy. Clin Ther. 2015;37(8):1813-21 e1. | |
|  | Downey, 2006 |
| Finitsis DJ, Pellowski JA, Johnson BT. Text message intervention designs to promote adherence to antiretroviral therapy (ART): a meta-analysis of randomized controlled trials. PLoS One. 2014;9(2):e88166. | |
|  | Musser, 2001 |
| Fenerty SD, West C, Davis SA, Kaplan SG, Feldman SR. The effect of reminder systems on patients' adherence to treatment. Patient Prefer Adherence. 2012;6:127-35. | |
|  | Armstrong, 2009 |
|  | Bender, 2010 |
|  | Cococila, 2009 |
|  | Fulmer, 1999 |
|  | Yentzer, 2011 |
| Fletcher BR, Hartmann-Boyce J, Hinton L, McManus RJ. The Effect of Self-Monitoring of Blood Pressure on Medication Adherence and Lifestyle Factors: A Systematic Review and Meta-Analysis. Am J Hypertens. 2015;28(10):1209-21. | |
|  | Bailey, 1999 |
|  | Bove, 2013 |
|  | Green, 2014 |
|  | Hosseininasab, 2014 |
|  | Johnson, 1978 |
|  | Magid, 2011 |
|  | McKenney, 1992 |
|  | Rudd, 2004 |
|  | Stewart, 2014a |
|  | Zarnke, 1997 |
| van Galen KA, Nellen JF, Nieuwkerk PT. The Effect on Treatment Adherence of Administering Drugs as Fixed-Dose Combinations versus as Separate Pills: Systematic Review and Meta-Analysis. AIDS Res Treat. 2014;2014:967073. | |
|  | Asplund, 1984 |
|  | Eron, 2000 |
|  | Geiter, 1987 |
|  | RCTAI, 1989 |
| Chase JA, Bogener JL, Ruppar TM, Conn VS. The Effectiveness of Medication Adherence Interventions Among Patients With Coronary Artery Disease: A Meta-analysis. J Cardiovasc Nurs. 2016;31(4):357-66. | |
|  | Campbell, 1998 |
|  | Costa, 2008 |
|  | Faulkner, 2000 |
|  | Gould, 2011 |
|  | Guthrie, 2007 |
|  | Kelly, 1988 |
|  | Kotowycz, 2010 |
|  | Lehr, 1986 |
|  | Lourenco, 2011 |
|  | Miller, 1990 |
|  | Muniz, 2010 |
|  | Nicoleau, 1985 |
|  | Shemesh, 2006 |
|  | Shemesh, 2006 |
|  | Sherrard, 2009 |
|  | Smith, 2008 |
|  | Yilmaz, 2005 |
|  | Zhao, 2004 |
| Caldeira D, Vaz-Carneiro A, Costa J. The impact of dosing frequency on medication adherence in chronic cardiovascular disease: systematic review and meta-analysis. Rev Port Cardiol. 2014;33(7-8):431-7. | |
|  | Andrejak, 2000 |
|  | Lee, 1996 |
| De Simoni A, Hardeman W, Mant J, Farmer AJ, Kinmonth AL. Trials to improve blood pressure through adherence to antihypertensives in stroke/TIA: systematic review and meta-analysis. J Am Heart Assoc. 2013;2(4):e000251. | |
|  | Maasland, 2007 |
| Kanters S, Park JJ, Chan K, Ford N, Forrest J, Thorlund K, et al. Use of peers to improve adherence to antiretroviral therapy: a global network meta-analysis. J Int AIDS Soc. 2016;19(1):21141. | |
|  | Altice, 2007 |
|  | Berg, 2011 |
|  | Gross, 2009 |
|  | Gross, 2015 |
|  | Macalino, 2007 |
|  | Nachega, 2010 |
|  | Ruiz, 2010 |

1. **Risk of bias assessment**


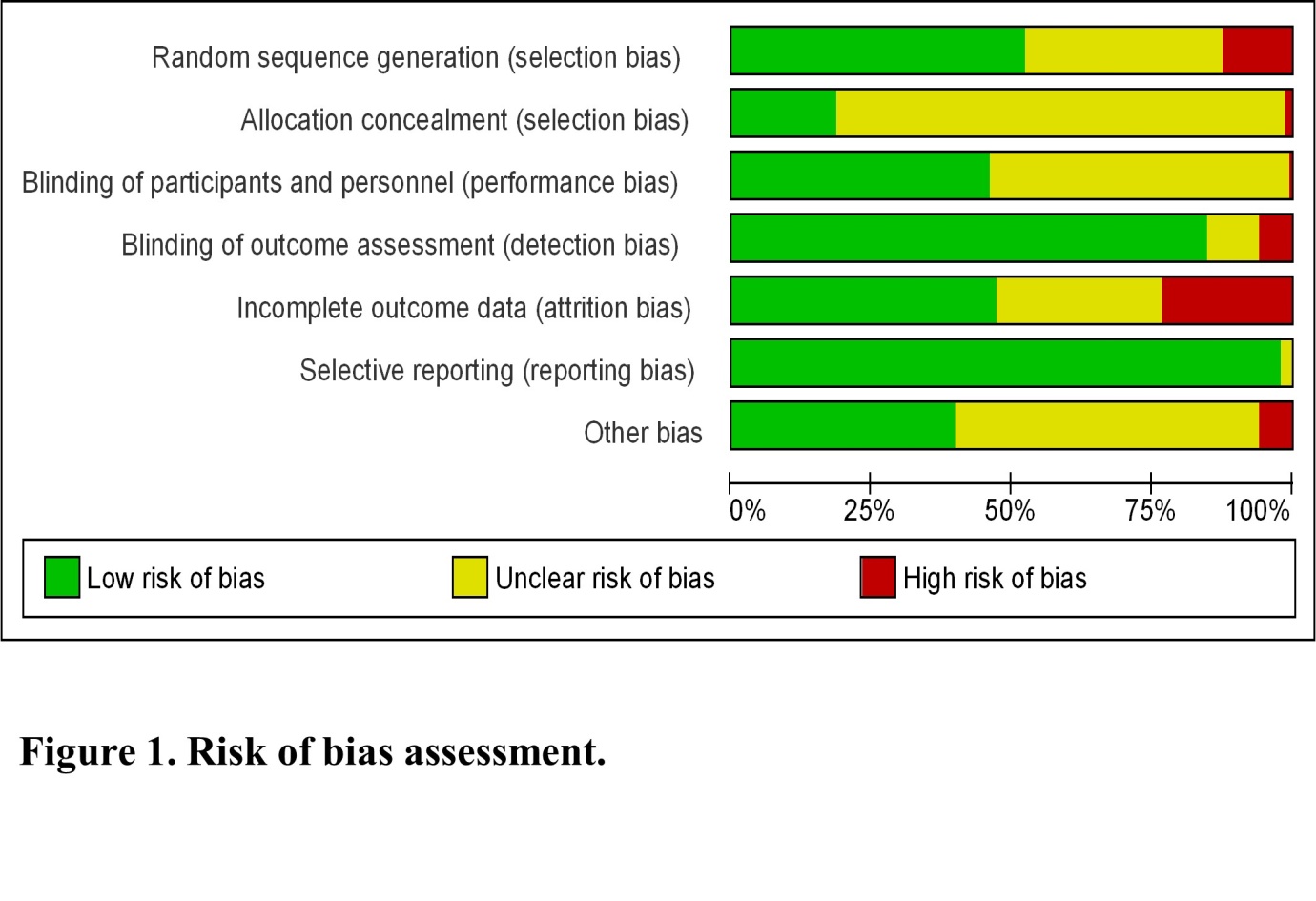


**Figure 1. Risk of bias assessment.**


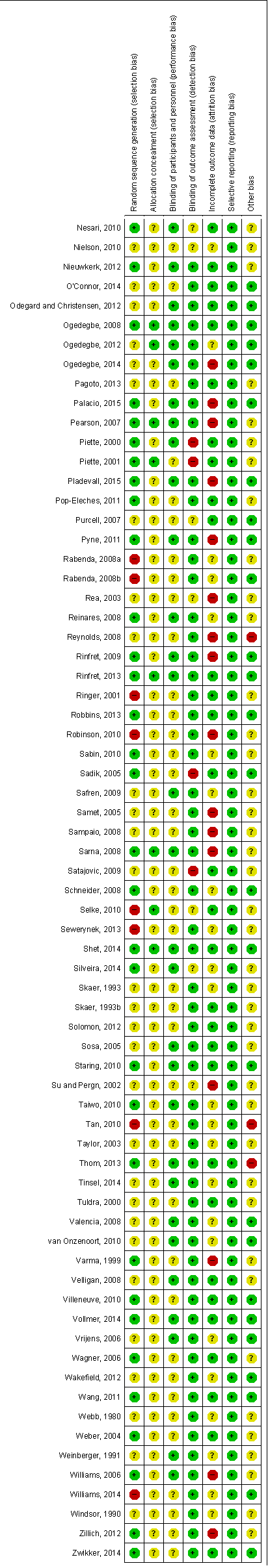

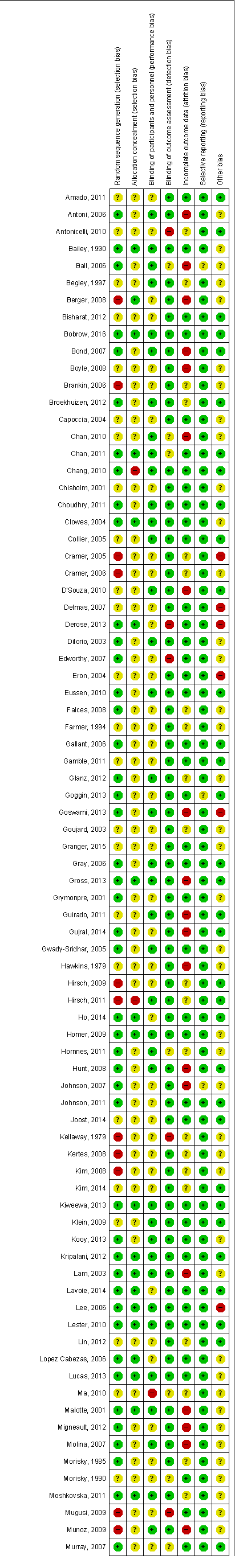


1. **Studies included in the network meta-analysis**
2. Cardiovascular and metabolic diseases

| **Study ID** | **Title** | **Study size** | **Interventions** |
| --- | --- | --- | --- |
| Antonicelli, 2010 | Impact of Home Patient Telemonitoring on Use of ß-Blockers in Congestive Heart Failure | 57 | Educational + Technical 4th, Standard care 4th |
| Broekhuizen, 2012 | Can Multiple Lifestyle Behaviours Be Improved in People with Familial Hypercholesterolemia? Results of a Parallel Randomised Controlled Trial | 224 | Educational + Attitudinal 4th, Standard care 4th |
| Choudhry, 2011 | Full Coverage for Preventive Medications after Myocardial Infarction | 5855 | Rewards 4th, Standard care 4th |
| Derose, 2013 | Automated Outreach to Increase Primary Adherence to Cholesterol-Lowering Medications | 5216 | Educational + Technical 4th, Standard care 4th |
| Edworthy, 2007 | Effects of an enhanced secondary prevention program for patients with heart disease: A prospective randomized trial | 2643 | Educational 4th, Standard care 4th |
| Eussen, 2010 | A Pharmaceutical Care Program to Improve Adherence to Statin Therapy: A Randomized Controlled Trial | 899 | Educational 4th, Standard care 4th |
| Falces, 2008 | [An educative intervention to improve treatment compliance and to prevent readmissions of elderly patients with heart failure] | 103 | Educational 4th, Standard care 4th |
| Goswami, 2013 | Impact of an integrated intervention program on atorvastatin adherence: a randomized controlled trial | 208 | Educational 4th, Standard care 4th |
| Gurjal, 2014 | Impact of community pharmacist intervention discussing patients’ beliefs to improve medication adherence | 200 | Attitudinal 4th, Standard care 4th |
| Hawkins, 1979 | Evaluation of a clinical pharmacist in caring for hypertensive and diabetic patients | 137 | Educational 4th, Standard care 4th |
| Ho, 2014 | Multifaceted Intervention to Improve Medication Adherence and Secondary Prevention Measures After Acute Coronary Syndrome Hospital Discharge A Randomized Clinical Trial | 241 | Educational + Technical 4th, Standard care 4th |
| Hornnes, 2011 | Blood Pressure 1 Year after Stroke: The Need to Optimize Secondary Prevention | 293 | Educational + Technical 4th, Standard care 4th |
| Hunt, 2008 | A Randomized Controlled Trial of Team-Based Care: Impact of Physician-Pharmacist Collaboration on Uncontrolled Hypertension | 272 | Educational + Technical 4th, Standard care 4th |
| Kooy, 2013 | Does the use of an electronic reminder device with or without counseling improve adherence to lipid-lowering treatment? The results of a randomized controlled trial | 381 | Technical 4th, Attitudinal + Technical 4th, Standard care 4th |
| Lopez Cabezas, 2006 | Randomized clinical trial of a postdischarge pharmaceutical care program vs. regular follow-up in patients with heart failure | 63 | Educational 4th, Standard care 4th |
| Morisky, 1985 | Evaluation of family health education to build social support for long-term control of high blood pressure. | 290 | Educational 4th, Standard care 4th |
| Ogedegbe, 2008 | A Practice-based Trial of Motivational Interviewing and Adherence in Hypertensive 065African Americans | 160 | Attitudinal 4th, Standard care 4th |
| Ogedegbe, 2012 | A Randomized Controlled Trial of Positive-Affect Intervention and Medication Adherence in Hypertensive African Americans | 256 | Educational 4th, Educational + Attitudinal 4th |
| Pagoto, 2013 | Can attention control conditions have detrimental effects in behavioral medicine randomized trials? | 235 | Educational 4th, Standard care 4th |
| Palacio, 2015 | Can Phone-Based Motivational Interviewing Improve Medication Adherence to Antiplatelet Medications After a Coronary Stent Among Racial Minorities? A Randomized Trial | 339 | Attitudinal 4th, Educational 4th |
| Piette, 2000 | Do Automated Calls with Nurse Follow-up Improve Self-Care and Glycemic Control among Vulnerable Patients with Diabetes? | 248 | Educational + Technical 4th, Standard care 4th |
| Piette, 2001 | Impact of Automated Calls With Nurse Follow-Up on Diabetes Treatment Outcomes in a Department of Veterans Affairs Health Care System A randomized controlled trial | 272 | Educational + Technical 4th, Standard care 4th |
| Rinfret, 2013 | Telephone contact to improve adherence to dual antiplatelet therapy after drug-eluting stent implantation | 300 | Educational 4th, Standard care 4th |
| Sadik, 2005 | Pharmaceutical care of patients with heart failure | 208 | Educational + Technical 4th, Standard care 4th |
| Taylor, 2003 | Improving primary care in rural Alabama with a pharmacy initiative | 69 | Educational + Technical 4th, Standard care 4th |
| Thom, 2013 | Effects of a Fixed-Dose Combination Strategy on Adherence and Risk Factors in Patients With or at High Risk of CVD The UMPIRE Randomized Clinical Trial | 1860 | Technical 4th, Standard care 4th |
| Varma, 1999 | Pharmaceutical Care of Patients with Congestive Heart Failure: Interventions and Outcomes | 49 | Educational + Technical 4th, Standard care 4th |
| Villeneuve, 2010 | A cluster randomized controlled Trial to Evaluate an Ambulatory primary care Management program for patients with dyslipidemia: the TEAM study | 225 | Educational 4th, Standard care 4th |
| Vollmer, 2014 | Improving Adherence to Cardiovascular Disease Medications With Information Technology | 21752 | Technical 4th, Educational + Technical 4th, Standard care 4th |
| Wang, 2011 | Effects of pharmaceutical care interventions on blood pressure and medication adherence of patients with primary hypertension in China | 59 | Educational + Technical 4th, Standard care 4th |
| Zillich, 2012 | Evaluation of Specialized Medication Packaging Combined With Medication Therapy Management: Adherence, Outcomes, and Costs Among Medicaid Patients | 14621 | Educational 4th, Standard care 4th |

1. HIV

| **Study ID** | **Title** | **Study size** | **Interventions** |
| --- | --- | --- | --- |
| Boyle, 2008 | Randomization to Once-Daily Stavudine Extended Release/Lamivudine/Efavirenz Versus a More Frequent Regimen Improves Adherence While Maintaining Viral Suppression | 300 | Technical 4th, Standard care 4th |
| Chang, 2010 | Effect of Peer Health Workers on AIDS Care in Rakai, Uganda: A Cluster-Randomized Trial | 1203 | Educational + Technical 4th, Standard care 4th |
| Collier, 2005 | A Randomized Study of Serial Telephone Call Support to Increase Adherence and Thereby Improve Virologic Outcome in Persons Initiating Antiretroviral Therapy | 101 | Educational + Attitudinal 4th, Standard care 4th |
| Eron, 2004 | Once-Daily versus Twice-Daily Lopinavir/Ritonavir in Antiretroviral-Naive HIV-Positive Patients: A 48-Week Randomized Clinical Trial | 38 | Technical 4th, Standard care 4th |
| Gallant, 2006 | Tenofovir DF, Emtricitabine, and Efavirenz vs. Zidovudine, Lamivudine, and Efavirenz for HIV | 509 | Technical 4th, Standard care 4th |
| Gross, 2013 | Managed Problem Solving for Antiretroviral Therapy Adherence: A Randomized Trial | 180 | Educational + Attitudinal + Technical 4th, Standard care 4th |
| Hirsch, 2009 | Evaluation of the First Year of a Pilot Program in Community Pharmacy: HIV/AIDS Medication Therapy Management for Medi-Cal Beneficiaries | 7018 | Educational 4th, Standard care 4th |
| Hirsch, 2011 | Antiretroviral Therapy Adherence, Medication Use, and Health Care Costs During 3 Years of a Community Pharmacy Medication Therapy Management Program for Medi-Cal Beneficiaries with HIV/AIDS | 2234 | Educational + Technical 4th, Standard care 4th |
| Johnson, 2011 | Improving Coping Skills for Self-management of Treatment Side Effects Can Reduce Antiretroviral Medication Nonadherence among People Living with HIV | 249 | Educational + Attitudinal 4th, Standard care 4th |
| Kiweewa, 2013 | Noninferiority of a Task-Shifting HIV Care and Treatment Model Using Peer Counselors and Nurses Among Ugandan Women Initiated on ART: Evidence From a Randomized Trial | 85 | Educational 4th, Standard care 4th |
| Lester, 2010 | Effects of a mobile phone short message service on antiretroviral treatment adherence in Kenya (WelTel Kenya1): a randomised trial | 538 | Technical 4th, Standard care 4th |
| Lucas, 2013 | Directly Administered Antiretroviral Therapy for HIVInfected Individuals in Opioid Treatment Programs: Results from a Randomized Clinical Trial | 107 | Technical 4th, Standard care 4th |
| Molina, 2007 | A Lopinavir/Ritonavir-Based Once-Daily Regimen Results in Better Compliance and Is Non-inferior to a Twice-Daily Regimen Through 96 Weeks | 190 | Technical 4th, Standard care 4th |
| Mugusi, 2009 | Enhancing adherence to antiretroviral therapy at the HIV clinic in resource constrained countries; the Tanzanian experience | 621 | Technical 4th, Educational + Technical 4th, Standard care 4th |
| Munoz, 2009 | Community-based DOT-HAART Accompaniment in an Urban Resource-Poor Setting | 120 | Educational + Technical 4th, Standard care 4th |
| Pearson, 2007 | Randomized Control Trial of Peer-Delivered, Modified Directly Observed Therapy for HAART in Mozambique | 350 | Educational + Technical 4th, Standard care 4th |
| Pop-Eleches, 2011 | Mobile phone technologies improve adherence to antiretroviral treatment in a resource-limited setting: a randomized controlled trial of text message reminders | 428 | Technical 4th, Standard care 4th |
| Purcell, 2007 | Results From a Randomized Controlled Trial of a Peer-Mentoring Intervention to Reduce HIV Transmission and Increase Access to Care and Adherence | 408 | Attitudinal 4th, Educational 4th |
| Pyne, 2011 | Effectiveness of Collaborative Care for Depression in Human Immunodeficiency Virus Clinics | 178 | Educational + Technical 4th, Standard care 4th |
| Reynolds, 2008 | Telephone Support to Improve Antiretroviral Medication Adherence | 109 | Educational 4th, Educational + Attitudinal 4th |
| Sabin, 2010 | Using Electronic Drug Monitor Feedback to Improve Adherence to Antiretroviral Therapy Among HIV-Positive Patients in China | 64 | Educational + Technical 4th, Standard care 4th |
| Samet, 2005 | A randomized controlled trial to enhance antiretroviral therapy adherence in patients with a history of alcohol problems | 94 | Educational + Attitudinal + Technical 4th, Standard care 4th |
| Selke, 2010 | Task-Shifting of Antiretroviral Delivery From Health Care Workers to Persons Living With HIV/AIDS: Clinical Outcomes of a Community-Based Program in Kenya | 208 | Educational 4th, Standard care 4th |
| Shet, 2014 | Effect of mobile telephone reminders on treatment outcome in HIV: evidence from a randomised controlled trial in India | 631 | Technical 4th, Standard care 4th |
| Silveira, 2014 | Randomized Controlled Trial to Evaluate the Impact of Pharmaceutical Care on Therapeutic Success in HIV-Infected Patients in Southern Brazil | 332 | Educational 4th, Standard care 4th |
| Sosa, 2005 | Abacavir and Lamivudine Fixed-Dose Combination Tablet | 236 | Technical 4th, Standard care 4th |
| Taiwo, 2010 | Assessing the Viorologic and Adherence Benefits of Patient-Selected HIV Treatment Partners in a Resource-limited Setting | 499 | Technical 4th, Standard care 4th |
| Tuldra, 2000 | Prospective Randomized Two-Arm Controlled Study To Determine the Efficacy of a Specific Intervention To Improve Long-Term Adherence to Highly Active Antiretroviral Therapy | 116 | Attitudinal 4th, Standard care 4th |
| Wagner, 2006 | Cognitive-behavioral intervention to enhance adherence to antiretroviral therapy: a randomized controlled trial (CCTG 578) | 199 | Attitudinal 4th, Standard care 4th |
| Weber, 2004 | Effect of individual cognitive behaviour intervention on adherence to antiretroviral therapy: prospective randomized trial | 60 | Attitudinal 4th, Standard care 4th |
| Williams, 2006 | Home Visits to Improve Adherence to Highly Active Antiretroviral Therapy: A Randomized Controlled Trial | 171 | Educational + Attitudinal 4th, Standard care 4th |
| Williams, 2014 | Efficacy of an Evidence-Based ARV Adherence Intervention in China | 110 | Educational + Attitudinal 4th, Standard care 4th |
|  |  |  |  |

1. Musculoskeletal diseases

| **Study ID** | **Title** | **Study size** | **Interventions** |
| --- | --- | --- | --- |
| Brankin, 2006 | The impact of dosing frequency on compliance and persistence with bisphosphonates among postmenopausal women in the UK: evidence from three databases | 15330 | Technical 4th, Standard care 4th |
| Clowes, 2004 | The Impact of Monitoring on Adherence and Persistence with Antiresorptive Treatment for Postmenopausal Osteoporosis: A Randomized Controlled Trial | 48 | Technical 4th, Standard care 4th |
| Cramer, 2005 | Compliance and persistence with bisphosphonate dosing regimens among women with postmenopausal osteoporosis | 2741 | Technical 4th, Standard care 4th |
| Cramer, 2006 | The Effect of Dosing Frequency on Compliance and Persistence with Bisphosphonate Therapy in Postmenopausal Women: A Comparison of Studies in the United States, the United Kingdom, and France | 15640 | Technical 4th, Standard care 4th |
| Delmas, 2007 | Effect of Monitoring Bone Turnover Markers on Persistence with Risedronate Treatment of Postmenopausal Osteoporosis | 2302 | Technical 4th, Standard care 4th |
| Homer, 2009 | Providing patients with information about disease-modifying antirheumatic drugs: Individually or in groups? A pilot randomized controlled trial comparing adherence and satisfaction | 62 | Educational 4th, Educational + Technical 4th |
| Nielson, 2010 | Patient education in groups increases knowledge of osteoporosis and adherence to treatment: A two-year randomized controlled trial | 300 | Educational 4th, Standard care 4th |
| Rabenda, 2008a | Adherence to bisphosphonates therapy and hip fracture risk in osteoporotic women | 29157 | Technical 4th, Standard care 4th |
| Rabenda, 2008b | Low Incidence of Anti-Osteoporosis Treatment After Hip Fracture | 306 | Technical 4th, Standard care 4th |
| Soloman, 2012 | Osteoporosis Telephonic Intervention to Improve Medication Adherence (OPTIMA): A Large Pragmatic Randomized Controlled Trial | 2087 | Attitudinal 4th, Educational 4th |
| Zwikker, 2014 | Effectiveness of a group-based intervention to change medication beliefs and improve medication adherence in patients with rheumatoid arthritis: A randomized controlled trial | 123 | Attitudinal 4th, Educational 4th |

1. Psychological diseases

| **Study ID** | **Title** | **Study size** | **Interventions** |
| --- | --- | --- | --- |
| Ball, 2006 | A Randomized Controlled Trial of Cognitive Therapy for Bipolar Disorder: Focus on Long-Term Change | 52 | Attitudinal 4th, Standard care 4th |
| Capoccia, 2004 | Randomized trial of pharmacist interventions to improve depression care and outcomes in primary care | 74 | Educational + Technical 4th, Standard care 4th |
| Lam, 2003 | A Randomized Controlled Study of Cognitive Therapy for Relapse Prevention for Bipolar Affective Disorder | 103 | Attitudinal 4th, Standard care 4th |
| Pyne, 2011 | Effectiveness of Collaborative Care for Depression in Human Immunodeficiency Virus Clinics | 178 | Educational + Technical 4th, Standard care 4th |
| Reinares, 2008 | Impact of caregiver group psychoeducation on the course and outcome of bipolar patients in remission: a randomized controlled trial | 113 | Educational + Attitudinal 4th, Standard care 4th |
| Silveira, 2014 | Randomized Controlled Trial to Evaluate the Impact of Pharmaceutical Care on Therapeutic Success in HIV-Infected Patients in Southern Brazil | 332 | Educational 4th, Standard care 4th |
| Valencia, 2008 | A psychosocial skills training approach in Mexican out-patients with schizophrenia | 82 | Educational 4th, Standard care 4th |
| Velligan, 2008 | The Use of Individually Tailored Environmental Supports to Improve Medication Adherence and Outcomes in Schizophrenia | 61 | Attitudinal 4th, Standard care 4th |
| Williams, 2014 | Efficacy of an Evidence-Based ARV Adherence Intervention in China | 110 | Educational + Attitudinal 4th, Standard care 4th |
| Zillich, 2012 | Evaluation of Specialized Medication Packaging Combined With Medication Therapy Management: Adherence, Outcomes, and Costs Among Medicaid Patients | 14621 | Educational 4th, Standard care 4th |

1. **Node-splitting analyses per disease group (Musculoskeletal and Psychological conditions do not have node-splitting analysis as this is only possible when there are close-loops in the networks)**
2. Cardiovascular and metabolic diseases

| **Name** | **Direct Effect** | **Indirect Effect** | **Overall** | **P-Value** |
| --- | --- | --- | --- | --- |
| Attitudinal, Educational | -0.61  (-2.22, 1.01) | 0.87  (-0.40, 2.19) | 0.29  (-0.77, 1.34) | 0.19 |
| Attitudinal, Standard care | 0.30  (-0.91, 1.49) | -1.15  (-2.87, 0.60) | -0.17  (-1.19, 0.87) | 0.19 |
| Educational, Educational + Attitudinal | 0.25  (-1.51, 1.93) | -0.60  (-2.44, 1.18) | -0.16  (-1.40, 1.06) | 0.6 |
| Educational, Standard care | -0.61  (-1.14, -0.08) | 0.68  (-0.86, 2.17) | -0.46  (-0.98, 0.06) | 0.9 |
| Educational + Attitudinal, Standard care | 0.10  (-1.64, 1.87) | -0.74  (-2.61, 1.02) | -0.31  (-1.56, 0.95) | 0.5 |
| Educational + Technical, Technical | -0.01  (-1.68, 1.60) | 0.57  (-1.21, 2.29) | 0.03  (-1.12, 1.16) | 0.6 |

1. HIV

| **Name** | **Direct Effect** | **Indirect Effect** | **Overall** | **P-Value** |
| --- | --- | --- | --- | --- |
| Attitudinal, Educational | -0.28  (-1.44, 0.89) | 0.04  (-0.97, 0.97) | -0.09  (-0.84, 0.60) | 0.66 |
| Attitudinal, Standard care | -0.32  (-1.07, 0.38) | -0.62  (-1.93, 0.71) | -0.40  (-1.00, 0.22) | 0.67 |
| Educational, Educational + Attitudinal | 0.53  (-1.71, 2.89) | 0.16  (-0.66, 1.02) | 0.17  (-0.56, 1.00) | 0.75 |
| Educational, Standard care | -0.37  (-1.00, 0.32) | -0.05  (-1.18, 1.12) | -0.31  (-0.84, 0.28) | 0.63 |
| Educational + Attitudinal, Standard care | -0.47  (-1.11, 0.15) | -0.77  (-3.30, 1.50) | -0.48  (-1.10, 0.11) | 0.8 |
| Educational + Technical, Technical | 0.76  (-0.96, 2.88) | -0.15  (-0.72, 0.42) | -0.10  (-0.64, 0.45) | 0.32 |

1. **SUCRA analyses per disease group**
2. Circulatory system and metabolic diseases


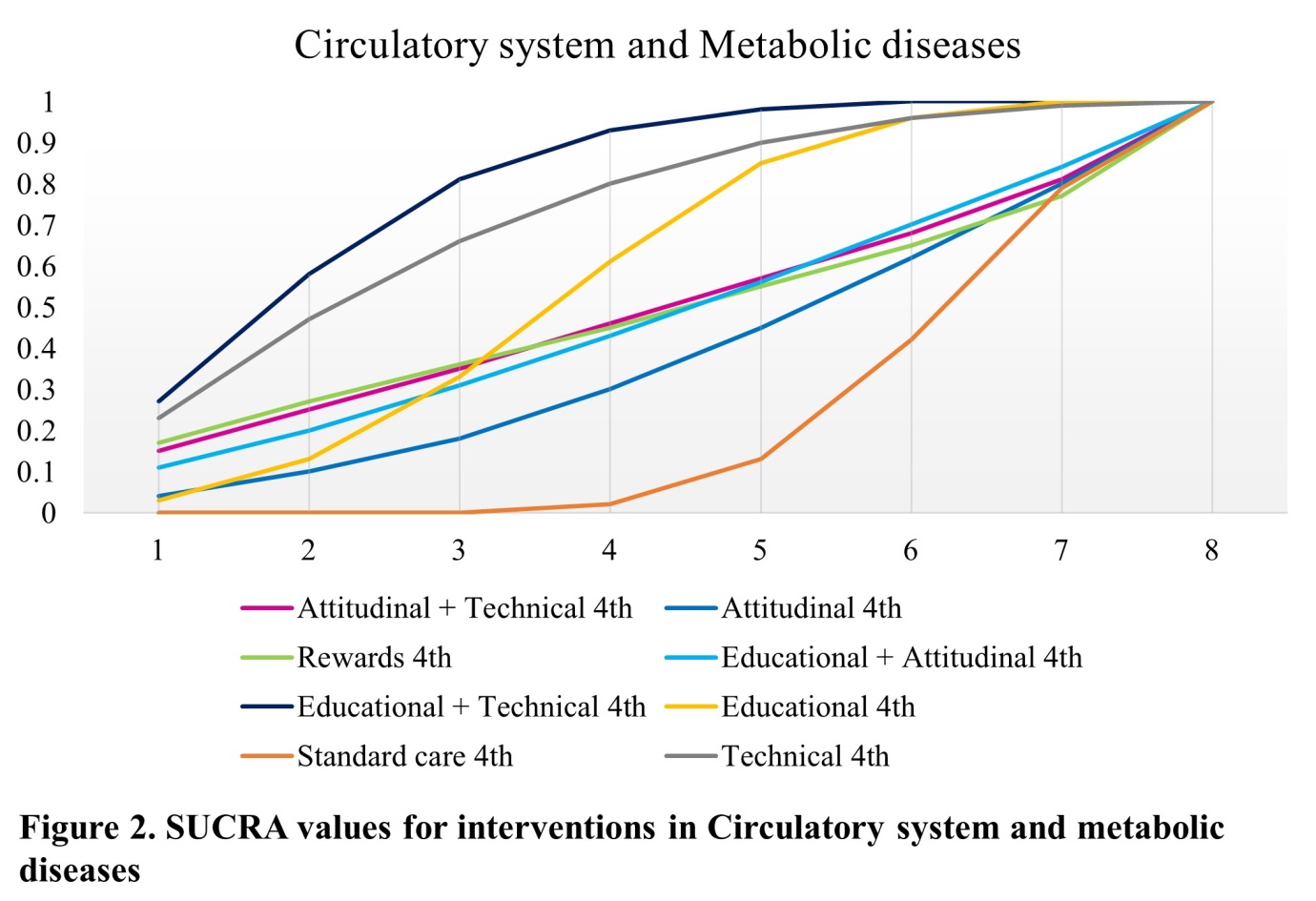


1. Infectious diseases (HIV)


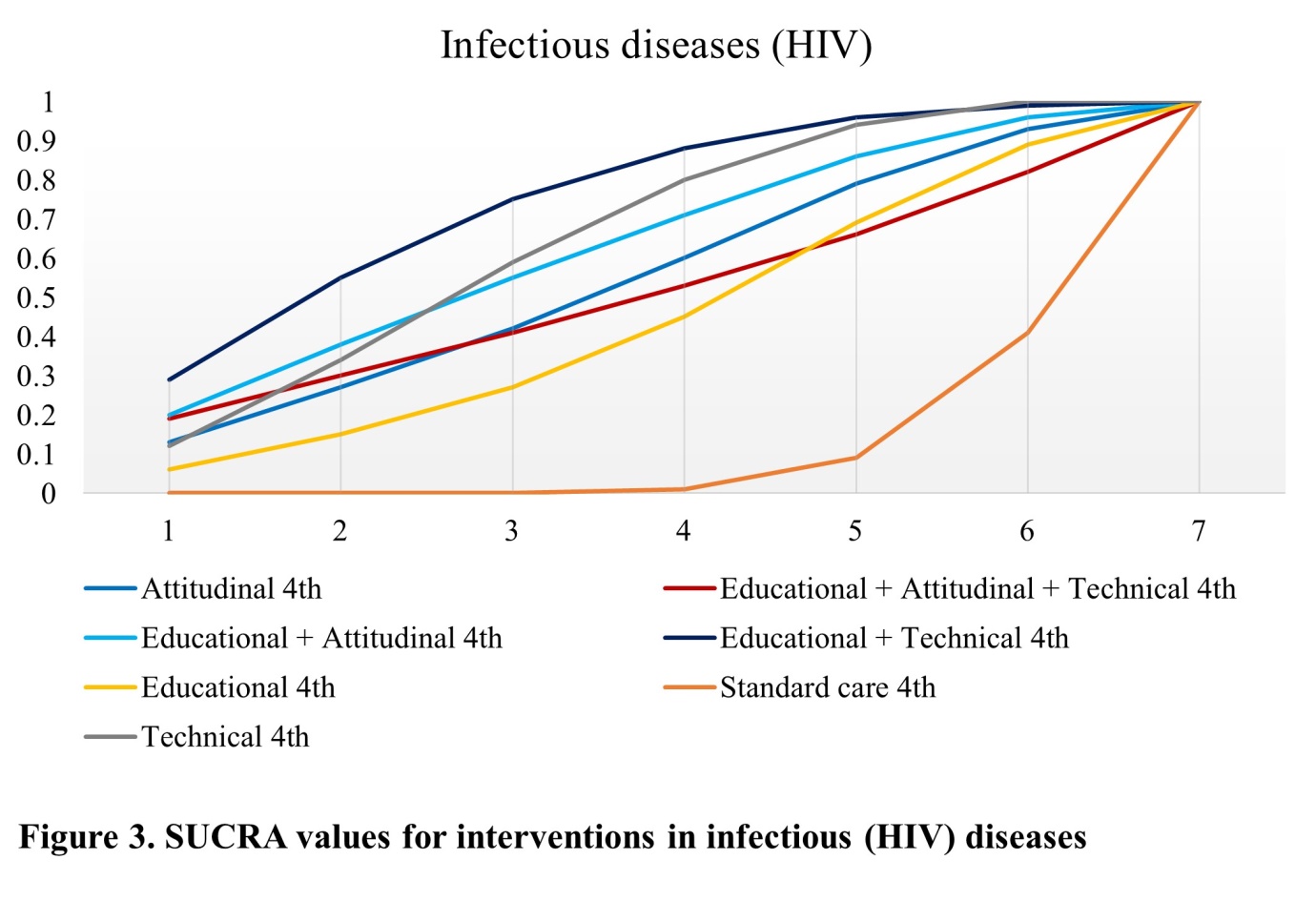


1. Musculoskeletal diseases


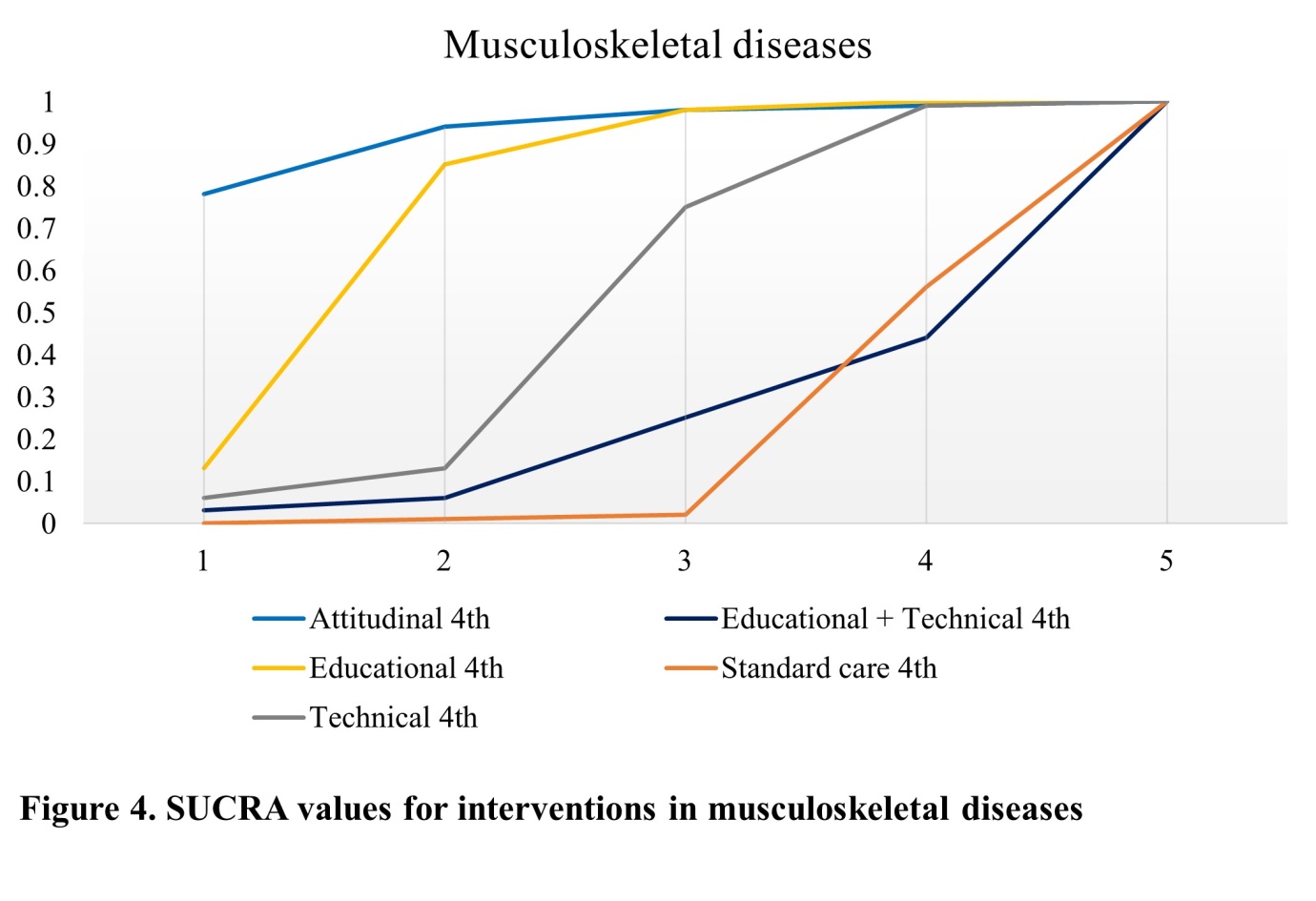


1. Mental, behavioural or neurodevelopmental disorders

**
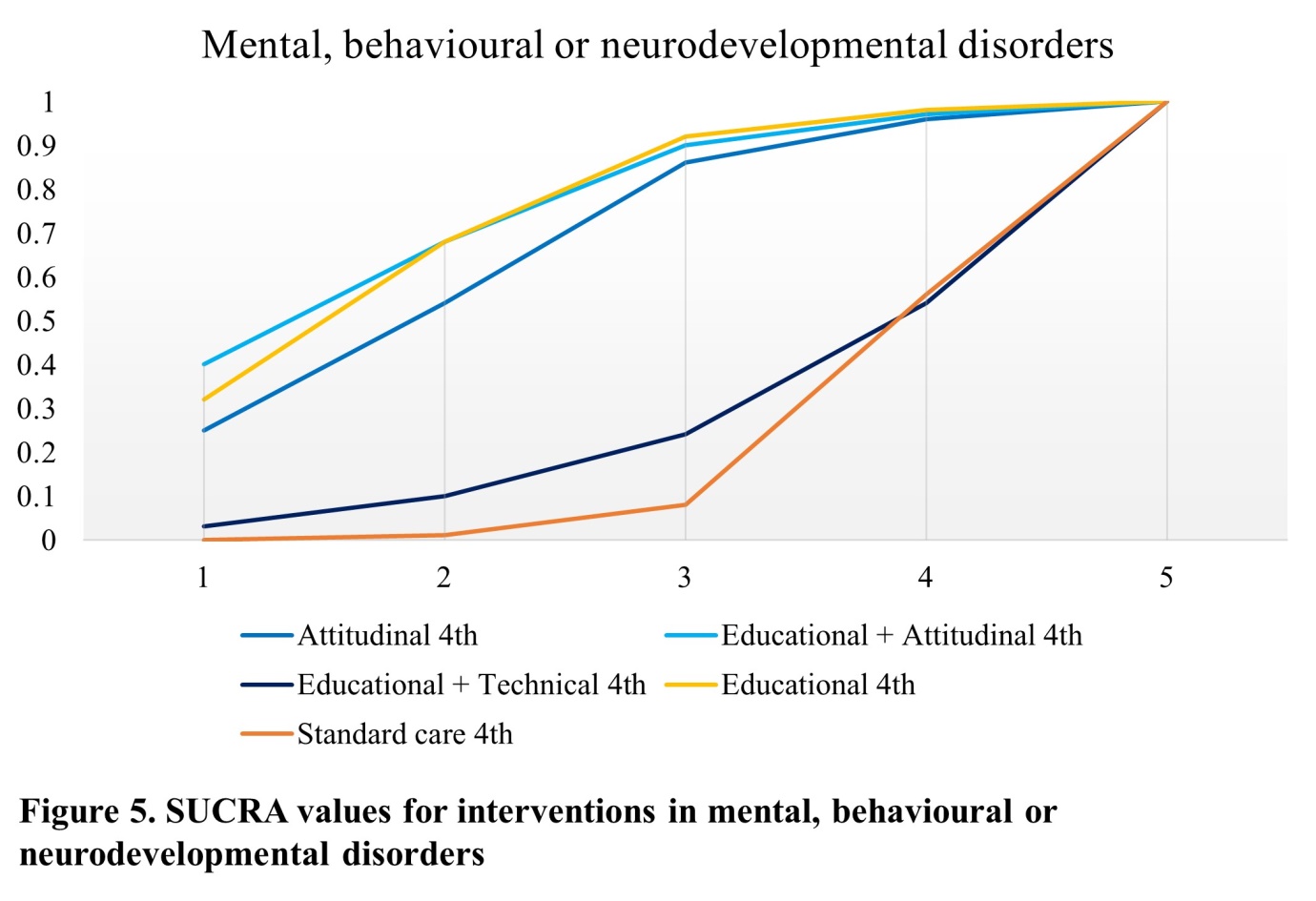
**
